# A systematic review and quality appraisal of the economic evaluations of schistosomiasis interventions

**DOI:** 10.1371/journal.pntd.0010822

**Published:** 2022-10-12

**Authors:** Sharon C. Uzoegbo, Louise J. Jackson, Sonja C. M. Bloch

**Affiliations:** Institute of Applied Health Research, University of Birmingham- College of Medical and Dental Sciences, Birmingham, United Kingdom; Royal Veterinary College Department of Pathology and Infectious Diseases: The Royal Veterinary College Department of Pathobiology and Population Sciences, UNITED KINGDOM

## Abstract

**Background:**

Schistosomiasis is a neglected tropical disease (NTD) that affects over 230 million people in low and middle-income countries (LMICs) and can lead to long-term debilitating health effects. It is associated with impoverishment and has been prioritised by the World Health Organization for prevention, control and elimination. This systematic review aimed to identify and evaluate existing economic evaluations of interventions to tackle schistosomiasis.

**Methodology:**

A comprehensive search strategy of four databases and additional hand-searching was employed on the 17^th^ July 2020. The articles were screened and sorted using a two-stage classification system. Full economic evaluations published in English between 1^st^ January 1998 and 17^th^ July 2020 were included, and methodological quality was appraised using the international decision support initiative (iDSI), Phillips and Evers checklists.

**Results:**

Eighteen economic evaluations were identified, nine trial-based and nine model-based, with the majority focused on preventative chemotherapy. Schistosomiasis interventions were collectively found to be cost-effective, but the quantity and quality of studies were limited. The outcome measures and time-horizons utilised varied substantially making comparison difficult. The majority of papers failed to address equity and affordability.

**Conclusion:**

Several methodological issues were highlighted which might have implications for optimal decision-making. Future research is needed to ensure the standardisation of methods, in order to ensure that scarce healthcare resources are focused on the most cost-effective programmes to tackle schistosomiasis and other NTDs.

## Author summary

Schistosomiasis is a neglected tropical disease that has been prioritised by the World Health Organization (WHO) for prevention, control and elimination. Its long-term devastating health effects are widely documented, as are its significant impacts on vulnerable communities. There is a deficit of donor funding for many schistosomiasis-endemic countries, and this paucity of financing requires affected states to adopt schistosomiasis interventions that are the most cost-effective, in order to maximise health outcomes for their populations.

This systematic review aims to identify and assess existing economic evaluations concerned with interventions to prevent, control, and eliminate schistosomiasis. We synthesise the existing evidence on cost-effectiveness and evaluate whether such studies are of acceptable methodological quality, and whether they are sufficiently transparent and reproducible. We conclude by highlighting methodological shortcomings in the current economic evidence base relating to schistosomiasis and make recommendations for the improvement of such studies.

This study contributes to the expanding body of evidence on economic evaluations of neglected tropical diseases (NTDs). The findings of this study will provide additional information for health authorities and policymakers in affected countries in the context of the identification of optimal programmes and interventions to tackle schistosomiasis.

## Introduction

Schistosomiasis, also known as bilharzia, is a neglected tropical disease (NTD) caused by a parasitic worm called a schistosome that infects human beings. It is a significant public health issue affecting low and middle-income countries (LMICs). More than 230 million people are infected with schistosomiasis globally, and close to 800 million are at risk of acquiring the disease [1]. Due to its substantial morbidity, the untreated disease causes significant long-lasting economic effects and causes an estimated 3.3 million disability-adjusted life years (DALYs) annually [1].

Untreated schistosomiasis in children can lead to chronic anaemias, stunted growth, and decreased learning capabilities. Poor childhood development can have lasting adverse effects into adulthood. Additionally, untreated chronic disease can lead to portal-hypertension, kidney disease, bladder cancers, and female genital schistosomiasis (FGS) which can manifest as chronic pelvic pain syndrome and infertility [2].

The World Health Organization (WHO) has prioritised schistosomiasis for elimination as a global public health problem. In 2012, a call was made for schistosomiasis-endemic member states of the WHO to work towards eliminating the disease [3]. Ending the NTD epidemics is further highlighted in the third Sustainable Development Goal (SDG), which focuses on the attainment of good health and wellbeing for all humans [4].

Humans become infected with the parasite when they come into contact with infested freshwater bodies. The parasite larvae enter the human by penetrating the skin barrier and migrating into the blood vessels. Inside the human host, they develop into an adult worm and reproduce, releasing eggs via the urine and faeces. The transmission cycle is maintained when humans infected with schistosomiasis urinate or defecate into freshwater bodies within which the intermediate host lives. Once the parasite’s eggs are released into the freshwater, they penetrate the snails and develop into immature forms within them, thus maintaining the transmission cycle [2].

Praziquantel is the drug of choice for the treatment and control of all five known species of schistosomiasis that infect humans [2]. Praziquantel is considered cost-effective by WHO and often given in a single one-off dose. Praziquantel cannot prevent re-infection with the parasite, and often more than a single dose will be required in endemic areas. Vaccine trials on animals have been ongoing for two decades, but currently, there is no vaccine approved for human use [1]. Prevention of the disease includes mass chemoprophylaxis in the susceptible and high-risk population groups in endemic areas, improvement of sanitation and hygiene, education and control of the snail vector [2].

Economic evaluations in healthcare are undertaken to improve the use of limited healthcare resources [5]. They have become an essential part of successful public-health programmes. At the end of the 1990s and in the early 2000s, the WHO-CHOICE (CHOosing Interventions that are Cost-Effective) along with investments of non-governmental and donor organisations, such as the Bill and Melinda Gates Foundation (BMGF), in economic evaluations, led to increasing attention towards conducting cost-effectiveness analyses (CEAs) in the LMIC context [5,6]. Lack of standardisation and discrepancies in reporting quality of economic evaluations is highlighted amongst the significant challenges faced in LMICs [5,7]. Poor reporting quality can translate into inaccurate results, which may have negative effects on public healthcare decisions.

Appropriate economic evidence is crucial for addressing NTDs, which are by their very definition conditions that have been historically neglected from a resource and economic investment viewpoint [8]. When analysing economic evaluations in many NTDs, additional considerations are required. Firstly, these diseases have a noticeable association with poverty, and a lack of intervention will result in the perpetuation of the poverty cycle. Poverty’s effect on families may create a ripple effect within their surrounding community, necessitating the need for the adoption of a societal perspective [2]. The societal perspective will encompass a broader picture of the socioeconomic impact of disease. Neglected tropical diseases can have a broad spectrum of outcomes, some of which can be difficult to quantify. This may be partly attributed to the vagueness of symptoms in infected individuals and the chronicity of untreated disease in many of the NTDs. Furthermore, there may be a long asymptomatic period, necessitating consideration of extended time-horizons. Additionally, NTDs occur in areas where disease notification and monitoring may be lacking, leading to inaccurate reporting of the disease burden [8]. NTDs have also been found to not occur in isolation. For example, studies have shown a geographical relationship and co-existence of schistosomiasis and soil transmitted helminths (STH) in schistosomiasis endemic areas [9]. This can create an opportunity to tackle both diseases simultaneously with a unified control programme.

This systematic review aims to identify and assess the economic literature that exists around interventions to prevent, control and eliminate schistosomiasis in LMICs. Systematic reviews on economic evaluations of schistosomiasis interventions currently exist, such as those by Garcia et al. [10] and Turner et. al [11], however their focus is not on methodological assessment. This review synthesises the existing evidence with the aid of economic evaluation checklists, and develops recommendations for the methodology of economic evaluations in this area, to help inform health policymaking in LMICs.

## Methods

A systematic review was undertaken to identify and evaluate the economic evidence on schistosomiasis interventions. The Preferred Reporting Items for Systematic Reviews and Meta-Analyses (PRISMA) guidelines were followed for reporting (S1 and S2 PRISMA Checklists) [12], and the Centre for Review and Dissemination (CRD) guidelines were adhered to for methods [13]. This systematic review was not registered, and the protocol was not published.

### Search strategy

A search strategy was developed for economic evaluations of schistosomiasis interventions. Four databases were searched: MEDLINE, EMBASE, Web of Science (WoS) and EconLit between 1998 and July 2020 (S6–S9 Tables). The year 1998 was chosen as a starting year, to reflect the development of the WHO-CHOICE project [6]. WHO-CHOICE has created recommendations for CEAs to help strengthen the standardisation of studies and establish minimally acceptable quality specifications [6]. A scoping search did not reveal any highly cited schistosomiasis full economic evaluations before this date. After selecting the final articles, a hand search of their reference lists was conducted to identify any additional eligible studies.

### Literature screening criteria

The PICO acronym (population, interventions, comparators, outcomes) was used to inform the search strategy and inclusion criteria. Studies that contained any intervention related to the prevention, treatment, control, elimination, interruption of disease transmission for schistosomiasis or a combination of any of the above were included. There were no restrictions for comparators in this review. The outcomes included were any that were health related. The study type was defined as a full economic evaluation (S1 Glossary).

The search and initial screening of articles was undertaken by SU. Search results from all databases were exported to an EndNote X9 folder, and the duplicates were removed. The remaining studies were categorised by SU independently and then checked by LJ independently by adapting an established three-stage system [14]. When additional advice was required, SB provided input.

Two stages were used in sorting. In Stage I, the studies were categorised into five groups A-E (Table 1). Group D and E studies were discarded. For Stage II, the studies in groups A, B and C were read in full and further classified into sub-groups (Table 1). The studies grouped as A(1,2) and B(1,2) were included in the quality assessment and narrative synthesis. All papers classified under sub-group 6 were discarded. The remaining studies [A(3–5) and B(3–5), all of category C] were used for additional background information.

**Table 1:** Categorisation of Studies.

**STAGE 1**	
A	Full economic evaluations of schistosomiasis interventions based on primary research
B	Full economic evaluations of schistosomiasis from studies based on information from differing sources, including economic models
C	Cost Analysis
D	Studies discussed any economic aspects of schistosomiasis but do not fit into category A, B or C
E	No relevance to economic evaluations in schistosomiasis
**STAGE 2**	
1.	Related to preventative or therapeutic chemotherapy alone
2.	Any combination of interventions that included snail control methods as a form of intervention
3.	Other interventions related to schistosomiasis not listed above
4.	Either a description of techniques but no working EE or social and economic related policy papers
5.	Systematic reviews
6.	Articles that are correspondence articles and supplementary articles

### Data extraction and synthesis

Data extraction took place using predefined data extraction tables [13]. Data relating to study characteristics, methodology, results, conclusions, and key assumptions were extracted. The data were tabulated and synthesised narratively. This is an appropriate method of synthesis for studies with methodological heterogeneity [12].

### Assessment of methodological quality

The methodological quality was assessed using the Consensus on Health Economic Criteria (CHEC) list for trial-based economic evaluations and the Philips checklist for modelling studies [15,16] as recommended by the Cochrane Handbook for Systematic Reviews [17]. All studies were further assessed with the international decision support initiative (iDSI) reference case (RC) for economic evaluations to ensure that the LMIC context was considered. The iDSI RC was initiated by the BMGF and is also known as the Gates reference case. The transition from the Gates RC to iDSI RC intended to create political neutrality and distance the RC from any private organisations [18]. The use of the iDSI reference case promotes standardisation to increase the transferability of economic evaluations in LMICs. Transferability greatly maximises the value and justifies the costs invested in the economic assessments conducted [18]. The results of the quality assessments were used to inform the analysis rather than to exclude studies.

## Results

**Fig 1:**
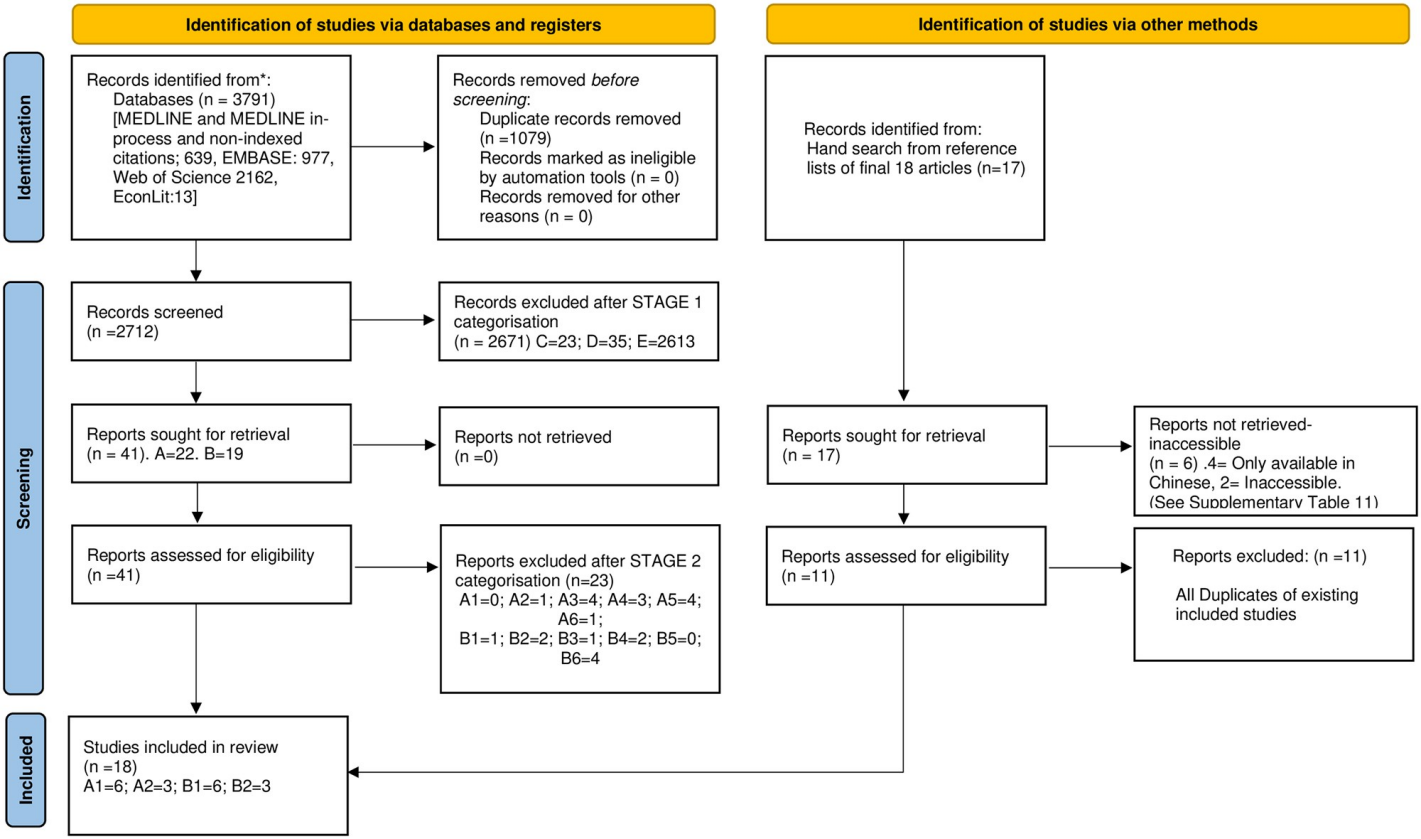
PRISMA flowchart depicting the process of study selection [12].

A systematic search yielded a total of 3808 articles. The electronic search found 3791 studies and hand searching identified a further 17 studies (Fig 1). From the hand search six papers were excluded due to inaccessibility, four were only available in Chinese and two were in English but inaccessible at the time of the search (S11 Table). The remaining 11 were duplicates of studies already included from the database search, consequently the hand search did not add any additional studies. After the removal of 1090 duplicates from the 3808 initial articles, the remaining 2712 articles were considered as part of Stage I of the categorisation process (screening of titles and abstracts). There were 41 papers taken for full-text examination and categorisation as part of Stage II. Of these 41 papers, 18 met all the inclusion criteria and were taken forward for quality assessment and synthesis.

### Study characteristics

Overall, 18 papers were identified which were concerned with the economic evaluation of interventions to prevent, control or eliminate schistosomiasis (Table 2). There were 11 studies which focussed on preventative chemotherapy (PC) as an intervention [19–29], six on integrated interventions involving a combination of chemotherapy [26,30–35], molluscicide and health education, and one hypothetical vaccination model. Praziquantel was the chemotherapy drug used in all the papers analysed. Four species of schistosoma were considered in the studies: *S*. *japonicum*, *S*. *haematobium*, *S*. *mansoni* and *S*. *mekongi*. Study settings were in 10 countries from Africa and Asia, with the highest number of studies concerned with China [29,30,34,35].

**Table 2:** Summarised Study Characteristics.

No.	Author (Year)	Parasite species	Intervention(s)	Comparator	Country setting (year)	Study population description (size)	Research question(s)	Ethical considerations	Type of Economic Evaluation
1	Yu et al. (2002) [29]	*S*. *japonicum*	Chemotherapy: 1. Mass 2. Screen 3.Clue	X	China (1998–2000)	3–65 year-olds from 8 villages around Dongting Lake (2615)	Which of the three interventions- clue, mass or screen chemotherapy is the most cost-effective choice for preventing schistosome infection and morbidity?	Ethical clearance and written participant consent obtained. Study objectives and benefits explained to community and community heads.	CEA
2	Leslie et al. (2011) [25]	*S*. *haematobium* and STH*	MDA in the community	MDA in schools	Niger (2004–2006)	4 communities and 8 schools in a highly-endemic area of Niger (530 000)	1.Which MDA is more cost-effective, school or community? 2.What are the costs involved in MDA? 3. What is the cost-per-infection treated? 4. What is the cost of delivering MDA through schools (via teachers) vs communities?	Ethical clearance obtained-written consent	CEA and Costing
3	Croce et al. (2010) [22]	*S*. *mekongi*	MDA in communities	Do-nothing approach	Cambodia (1995–2006)	Schistosoma endemic area in Cambodia	1. What is the cost-effectiveness of the MDA schistosomiasis control programme in Cambodia? 2. What is the cost-benefit of MDA schistosomiasis control programme in Cambodia from the perspective of the society?	The poor and marginalised communities benefited from free treatment	CEA and CBA
4	Carabin et al. (2000b) [21]	*S*. *mansoni*	Treatment of: 1.Symptomatic patients 2. Reporting blood in stool 3. Screening all symptomatic patients using a Kato-Katz smear and treating only the ones found positive	X	Burundi (1989–1990)	Burundi Rusizi Plain—all age groups who visited their PHCC with symptoms of *S*. *mansoni* (140 000)	What is the most cost-effective option for treating suspected *S*.*mansoni* following a patient history being suggestive of infection?	X	CEA
5	Brooker et al. (2008) [19]	Schistosoma and Intestinal nematodes	School-based MDA for STH and schistosomiasis	Do-nothing approach	Uganda (2003–2005)	School-going children Uganda (400000)	1.How does the cost and cost-effectiveness of a national school based STH and schistosomiasis control programme in Uganda differ regionally? 2. What are the main costs involved in the programme?	Ethically difficult to receive approval conduct a randomised control trial on the prevalence of anaemia cases on children to assess which were related to schistosomiasis as it would create ethical issues	Costing and CEA
6	Zhou et al. (2005) [35]	*S*. *japonicum*	Chinese national schistosomiasis programme-including MDA and mollusciciding	X	China (1992–2000)	6 counties in China	CEA and CBA of the national schistosomiasis control programme in China	X	CBA and CEA
7	Yu et al. (2013) [34]	*S*. *japonicum*	Comprehensive programme including environmental modifications, and WASH interventions	Standard programme which includes molluscicides, chemotherapy (animals and humans), patient education	China (2003–2006)	China- village around freshwater lake. 6–60 year-olds (ca. 4000)	What is the cost-effectiveness and effect of a schistosomiasis prevention program using the schistosomiasis comprehensive impact (SCI) index?	X	CEA and Impact
8	Guo et al. (2005) [30]	*S*. *japonicum*	Combination of passive chemotherapy and health education	Standard annual MDA	China (1998–2000)	China- endemic region, 6–65 year-olds (2 villages- 785 people)	What is the effect of replacing the standard MDA chemotherapy programme with a combination programme of passive chemotherapy and health education?	X	Impact and CEA
9	Guyatt et al. (2001) [24]	Schistosoma Hook worm	Chemotherapy	X	Tanzania-Zanzibar (1995–1997)	8–9 year-olds and 12–14 year-olds (466)	What is the impact of school-based deworming on anaemia cases in SAC in Tanzanian region?	Due to ethical reasons the children in each survey where different- ethical reasons not transparent	CEA
10	Collyer et al. (2019) [36]	*S*. *mansoni*	Hypothetical vaccination	MDA	X	X	What is the cost-effectiveness of a hypothetical schistosomiasis vaccination?	X	CEA
11	Lo et al. (2016) [27]	*S*. *haematobium S*. *mansoni*. [Ascaris lumbricoides, hookworm and Trichuris trichuria]	PC for STH and schistosomiasis	No treatment	Sub-Saharan Africa	X	At what prevalence thresholds is a combined programme PC programme of schistosomiasis and STH highly cost-effective?	X	CUA
12	Lo et al. (2015) [26]	*S*. *haematobium*. *S*. *mansoni*., STH*	1.MDA for only SAC 2.SAC and preschool-aged children 3.Adults alone (≥ 15yrs) 4.Whole community	No treatment	Côte d’Ivoire (1997–2010)	4 local communities in Ivory Coast (5000)	What is the cost-effectiveness of the various PC MDA approaches?	X	CUA
13	De Neve et al. (2018) [23]	Schistosomiasis [Lymphatic filariasis STH* (*Ascaris lumbricoides*, *Trichuris trichiura*, and hookworm infections)]	School-based MDA	Current status quo- no national intervention	Madagascar	5–14 year-olds (6 million)	What are the educational, financial and health benefits that can be achieved with the creation of a national NTD programme in Madagascar?	X	Extended-CEA/ CUA and CBA
14	Carabin et al.(2000a) [20]	*S*. *mansoni and S*. *haematobium*	1.School-going children and 25% school-aged children (SAC) that are not attending school 2.School-going children and 50% SAC 3.School-going children and 85% SAC	School- going children MDA	Egypt (1990)	6–15 year-olds (100 000)	Comparison of the cost-effectiveness of treating SAC vs only school-enrolled children.	Duty to provide treatment to children, not in school, who may be from even more impoverished backgrounds	CEA
15	Ndeffo-Mbah et al. (2013a) [33]	*S*. *haematobium* (and HIV)	SAC MDA- 2 scenarios 1. MDA reduces risk of HIV transmission 2. MDA reduces the prevalence of FGS	No MDA	Zimbabwe (2000)	15–49 year-olds (4 million)	Is MDA for treating schistosomiasis cost-effective in prevention of HIV in *S*. *haematobium* areas of endemicity?	X	CEA
16	Ndeffo-Mbah et al. (2013b) [28]	*S*. *haematobium* (and HIV)	MDA of chemotherapy distribution to SAC with community-wide WASH interventions	Status quo- No MDA, no WASH	Zimbabwe (20000)	(150000)	Is the combination of WASH interventions alongside treating SAC with MDA cost-effective as an intervention against HIV and *S*. *haematobium*?	X	CEA and CUA
17	Lo et al. (2018) [32]	*S*. *haematobium*	1.CWT with mollusciciding- LOW BURDEN VS HIGH BURDEN 2.Community-wide treatment (CWT), bi-annual and annual treatment	School-based MDA	Kenya	(5000)	What is the impact and cost-effectiveness of schistosomiasis interventions that involve mollusciciding either alone or in combination with other MDA, in areas of differing disease burden?	X	CEA and CUA
18	Kirigia (1998) [31]	Schistosoma	1.Focal mollusciciding 2. Household piped water supply 3. House-to-house health visits 4. Household vented improved pit latrine 5.Mass population chemotherapy with-praziquantel 6. Mass population chemotherapy with-oxamniquine 7. Selective population chemotherapy-praziquantel 8. Selective population chemotherapy-omniquine	Status quo	Kenya	X	What is the most cost-effective intervention for schistosomiasis control?	X	CUA

X—Not reported by the study.

*STH-Soil transmitted helminths—parasitic worms that affect humans and animals and are transmitted via contaminated soil.

CBA—Cost-benefit analysis, CEA—Cost-effectiveness analysis, CUA—Cost-utility analysis, CWT–Community wide treatment, HIV—Human immunodeficiency virus, MDA—Mass drug administration, N/S—Not stated, NGO—Non-governmental organisation, PC—Preventative chemotherapy, PHCC—Primary health care centre, SAC—School-aged children, STH—soil-transmitted helminths, WASH—Water, Sanitation and Hygiene

### Methodological considerations

#### Types of economic evaluation

Twelve of the papers were primarily CEAs [19–22,24,25,29,30,33–36]. Three studies conducted cost-benefit analyses (CBA) alongside either a (cost-utility analysis) CUA or CEA, which expressed the benefits in monetary values and utilised a benefit-cost ratio [22,23,35]. Six studies were CUAs [23,26–28,31,32] (see Table 2). Three of the CUA papers were combined with CEAs, as more than one metric was used to evaluate the outcome [23, 28, 32]. Nine studies involved a mathematical model [20,23,26–28,31–33,36] and the remaining nine were trial-based economic evaluations [19,21,22,24,25,29,30,34,35].

#### Study perspective

The perspective of the studies was mainly quasi-governmental and governmental organisational viewpoints, which were often not fully described (Table 3). Approximately 30% of papers did not clearly state their study perspective [20,24,25,30,34,35]. A societal perspective was not widely used, with only two papers adopting this approach [22, 31]. Kirigia [31] employed a societal perspective and Croce et al. [22] took productivity gains into consideration by adopting both a governmental and a societal perspective.

**Table 3:** Methodology.

No.	Author (year)	Type of modelling	Perspective	Time-horizon	Discounting	Costing	Cost-effectiveness threshold	Validation of Results	Sensitivity analysis	Outcome measure	Sub-Group Analysis
1	Yu et al. (2002) [29]	N/A	Healthcare-provider	Trial-period (1998–2002)	0% except microscope cost (10%)	Bottom-up costing Expert approximation for missing data items	A weighting system	Yes	▪Univariate	1.Cost-per-infection averted 2.Cost-per-1%-reduction of EPG 3. Cost-per-case of liver abnormality prevented 4. Cost per-case-of Spleen-abnormality prevented	X
2	Leslie et al. (2011) [25]	N/A	X	Trial-period (2004–2006)	3% costs	Extensive costing Economic costs	X	Yes, for costing aspects of study	▪Univariate ▪Scenario	Cost-per-infection averted	Adults and children (5–14 years)
3	Croce et al. (2010) [22]	N/A	Government and Societal	Trial-period (1995–2003)	X	Indirect and direct costs Economic costs	X	Yes. Compared to malaria prevention and measles vaccination programmes	▪Univariate	1.Cost-per-death averted 2. Cost per-severe infection averted 3. Cost-per-infection averted	X
4	Carabin et al. (2000b) [21]	N/A	Policymaker	Trial-period (1985–1989)	X	Unit-cost per-intervention per-child	X	Yes. Similar results obtained in Tanzania study.	▪Univariate	Number of infected patients treated	X
5	Brooker et al. (2008) [19]	N/A	Government	Trial-period (2003–2005)	3% capita costs	Only costs associated with treatment through a school-based distribution was considered Economic costs	X	Yes. Compare to study done in Tanzania, Burkina Faso and Ghana	▪Univariate ▪Scenario	Anaemia-cases averted	X
6	Zhou et al. (2005) [35]	N/A	X	Trial-period (1992–2000)	X	Top-down costing	X	X	X	**1:CEA** a)Cost of case-detected per individual b)Overall cost of project per individual c) Cost-per-1%-reduction in human infection rate per-100 individuals d) Cost per mollusciciding-to-reduce 1000m2 of snail-infested areas f) Cost to reduce-1% bovine-infection rate-per-100 cattle **2.CBA**: a) The amount of money gained per US$ 1 spent	X
7	Yu et al. (2013) [34]	N/A	X	Trial-period (2003–2006)	X	Unclear	X	X	X	1.Unit cost of reducing human *S*. *japonicum* infection by 1% 2.The Schistosomiasis Comprehensive Impact Index (SCI)	X
8	Guo et al. (2005) [30]	N/A	X	Trial-period (1998–2000)	X	The per diems and transport costs of the medical team were excluded	X	X	X	X	X
9	Guyatt et al. (2001) [24]	N/A	X	15 months	X	Unit-cost per-intervention per-child.	US$ 10 but not explicitly stated	Yes. Compared study costing with another Tanzanian study—explained reasons for differences	X	Per-anaemia case prevented	Two age-groups
10	Collyer et al. (2019) [36]	Dynamic	Healthcare provider	30 years	3% costs and effects	Obtained with WHO regression tool using median input values and mean GDP per capita of population needing treatment. Economic costs	Critical vaccination cost	X	▪Univariate ▪Scenario	High-intensity-infection-years averted	Population heterogeneity included in mathematical model
11	Lo et al. (2016) [27]	Adapted- Dynamic	National Preventative Chemotherapy Programme	5 years	3% costs and effects	Unit costs-Medication and Delivery of medication	US$ 1045 per DALY - GDP per-capita of a low-income country	Yes. Country specific scenario analysis—Kenya, Liberia, Ivory Coast	▪Univariate ▪Scenario	DALYs-averted	School children, pre-school children and adults
12	Lo et al. (2015) [26]	Adapted-Dynamic	National treatment programme	15 years	3% costs and effects	Indirect costs not included	Highly cost-effective if the ICER ≤Côte d’Ivoire annual GDP (US$ 1521)	Yes	▪Univariate ▪PSA	DALYs-averted	X
13	De Neve et al. (2018) [23]	Static-Own	Government	X	Wage benefits over 20 years with 3% discounting	Bottom-up	X	Yes	▪Univariate ▪Multiway ▪PSA	1.DALYs-averted 2.Out-of-pocket treatment costs averted 3.School absenteeism cases averted	Done- but not described
14	Carabin et al. (2000a) [20]	Dynamic	X	15 years (5 years intervention and 10 years without intervention)	5% effects	Unit-costing. Top-down	X	X	▪Univariate	1. Number of infections averted 2. Number of early-disease-cases averted	1)Yes. Male and female each had a school enrolled and non-school enrolled group. 2)Different species of Schistosomiasis had different analyses
15	Ndeffo-Mbah et al. (2013a) [33]	Dynamic-Own	Health-payers	10 years	3% costs and effects	Only direct medical costs-	X	Yes. Zimbabwe antenatal clinics for HIV prevalence. Validity of input estimates	▪PSA	Cost per HIV cases and medical care averted	X
16	Ndeffo-Mbah et al. (2013b) [28]	Dynamic	Health-payers	20 years	3% costs and effects	Only medical costs	Less than 3x Zimbabwean GDP very cost-effective. Less than 1x GDP effective	Costs—with other studies involving WASH in LMICs	▪PSA	1.DALYs 2.HIV-averted (20 years)	X
17	Lo et al. (2018) [32]	Adapted-Dynamic	National disease-control programme	10 years	3% costs and effects	MDA—bottom-up Molluscicide—top-down	ICER- HIGHLY CE if ICER < US$ 1,377 (Kenyan GDP)	Compared to studies in China- similar conclusions	▪Univariate ▪Multi-way ▪Scenario	DALYs	X
18	Kirigia (1998) [31]	Decision tree-Own	Societal	15 years	10% costs and effects	Direct, indirect and opportunity cost Economic costs	X	X	▪Univariate	QALYs	X

X—Not fulfilled by the study

Adapted—Adapted existing model, CBA—Cost-benefit analysis, CEA—Cost-effectiveness analysis, DALYs—Disability-adjusted life years, EPG—Eggs per gram of faeces, GDP—Gross domestic product, HIV—Human immunodeficiency virus, ICER—Incremental cost-effectiveness ratio, LMIC—Low- and middle-income country, Own—Created own model, PSA—Probabilistic sensitivity analysis, QALYs—Quality-adjusted life year US$-United States Dollar

Only three studies justified their chosen perspective [19,28,33]. Brooker et al. [19] explained that they had adopted a government perspective as their school-based MDA programme would only involve minimal costs to society. Ndeffo-Mbah et al. [28,33] cited health-payers (defined as national governments and donors), as the chosen perspective due to them being the primary providers of medical care related to human immunodeficiency virus (HIV) and schistosomiasis in sub-Saharan Africa (SSA).

#### Epidemiology and prevalence

The focus of the schistosomiasis interventions in over 50% of the studies were school-aged children (SAC) [19,20,23–28,32,36]. The SAC age range was approximately 5–14 years. Some of the SAC studies did additionally look at the cost effectiveness of treating adults and the whole community [26,27,32,36]. None of the studies based in China considered treatment according to age groups, with interventions being applied community wide. Mbah et al. [28] analysed MDA in SAC with WASH interventions that were applied community wide.

The authors noted the baseline prevalence rates used in their studies. They were based on region or country specific surveillance data that had been collected prior to the economic evaluation being undertaken or used in previous studies in the region. The hypothetical vaccination however used estimated prevalence rates, due to it not being a region-specific study. Collyer et al. [36] utilised the WHO information available on low and high transmission prevalence rates, and calculated the baseline prevalence by quantifying the rate of contact that exists between humans and the disease reservoir.

#### Discounting

The discount rates used ranged from 0% to 10% (Table 3). Only one-third of studies used a 3% discount rate for both costs and effects, in-line with WHO recommendations [6]. Three studies stated that they discounted only costs at a value of 3% [19,23,25].

#### Time-horizon

The time-horizon considered in the papers varied significantly from 15 months to 30 years. The period of collection of clinical and epidemiological data for trial-based economic evaluations restricted their time-horizon, from 15 months to nine years. The model-based economic evaluations ranged in time-horizons from 5 years to 30 years (Table 3). One model-based paper did not refer to the time-horizon adopted [30]. The authors cited various reasons for the different time-horizons adopted. For example, Collyer et al. [36] adopted a 30-year time-horizon to model a hypothetical vaccination, and the duration was explored in the sensitivity analysis.

#### Cost data

The papers identified essential cost components which fell into two broad categories: costs of medication and delivery costs. Three studies [23,29,32] specified bottom-up costing, whilst most employed a top-down approach. The trial-based studies primarily collected cost data prospectively utilising local databases, interviews with local government staff or utilisation of expert opinion. Model-based studies mainly derived their cost data from systematic reviews; five [26–28,32,33] of the nine economic evaluation models made use of a systematic review by King et al. [37] based on high-burden African countries.

Five papers utilised economic costs in their analysis [19,22,25,31,36], one paper was unclear [23], and the remaining papers only utilised financial costs. Economic costs take into consideration the opportunity cost which creates a more comprehensive representation of all costs involved [19]. The costing was generally not detailed in the papers. The individual drug values were mentioned in just under half of the papers [21,24,26–28,32,36], and individual drug costs (excluding administration costs) were noted to be under US$ 1 (0.08–0.99) in reviewed papers. The largest costs were centred around the running of the programmes themselves and payment of staff.

The WHO, in collaboration with the pharmaceutical industry, has created a donation scheme in which SSA countries with a high burden of schistosomiasis, can access free chemotherapy [2]. This donation is conditional on the country being able to provide evidence of a national strategy for control of the disease.

### Outcome measures

A range of outcome measures were used in the studies. Five studies utilised DALYs [23,26–28,32], and only one study utilised quality-adjusted life years (QALYs) [31]. The five studies utilising DALYs used published data from other papers, that were based on the 2010 Global Burden of Disease schistosomiasis DALY weightings. The other studies utilised a range of effectiveness measures, and five opted for the use of more than one effectiveness measure [20,22,29,34,35]. None of the trial-based economic evaluations used DALYs or QALYs as outcomes. Four papers utilising DALYs highlighted the shortcomings of the disability weights used in schistosomiasis as a limitation to their economic evaluation [23,26,27,32].

### Model type

For the nine model-based studies, dynamic models were the main type of model used (Table 3). De Neve et al. [23] and Kirigia [31] utilised static models, with only De Neve et al. acknowledging the limitations of their model type for schistosomiasis economic evaluations [23].

All the model-based papers discussed their assumptions (S1 Table), though some papers were more explicit and exhaustive than others when listing these. Four of the papers developed their mathematical transmission model de novo for the study [23, 28, 31, 33], with three providing information on the methodology used in the creation of their dynamic models. The other studies adapted pre-existing models from one or more published studies.

### Sensitivity analysis

The majority of papers specified the type of sensitivity analysis employed, which was largely univariate (Table 3). Sensitivity analysis was not discussed in four papers [24,30,34,35]. Only four studies utilised probabilistic sensitivity analysis (PSA) methods [23,26,28,33]. The most common input parameter analysed was the cost related to the interventions.

Most papers included univariate analysis of medication or vaccination costs alone, as part of their sensitivity analysis. This is important to note as certain countries are entitled to receive free chemotherapy [2], which can significantly alter the cost-effectiveness of their schistosomiasis prevention and treatment programmes.

## Appraisal of economic evaluations using quality checklists

**Fig 2:**
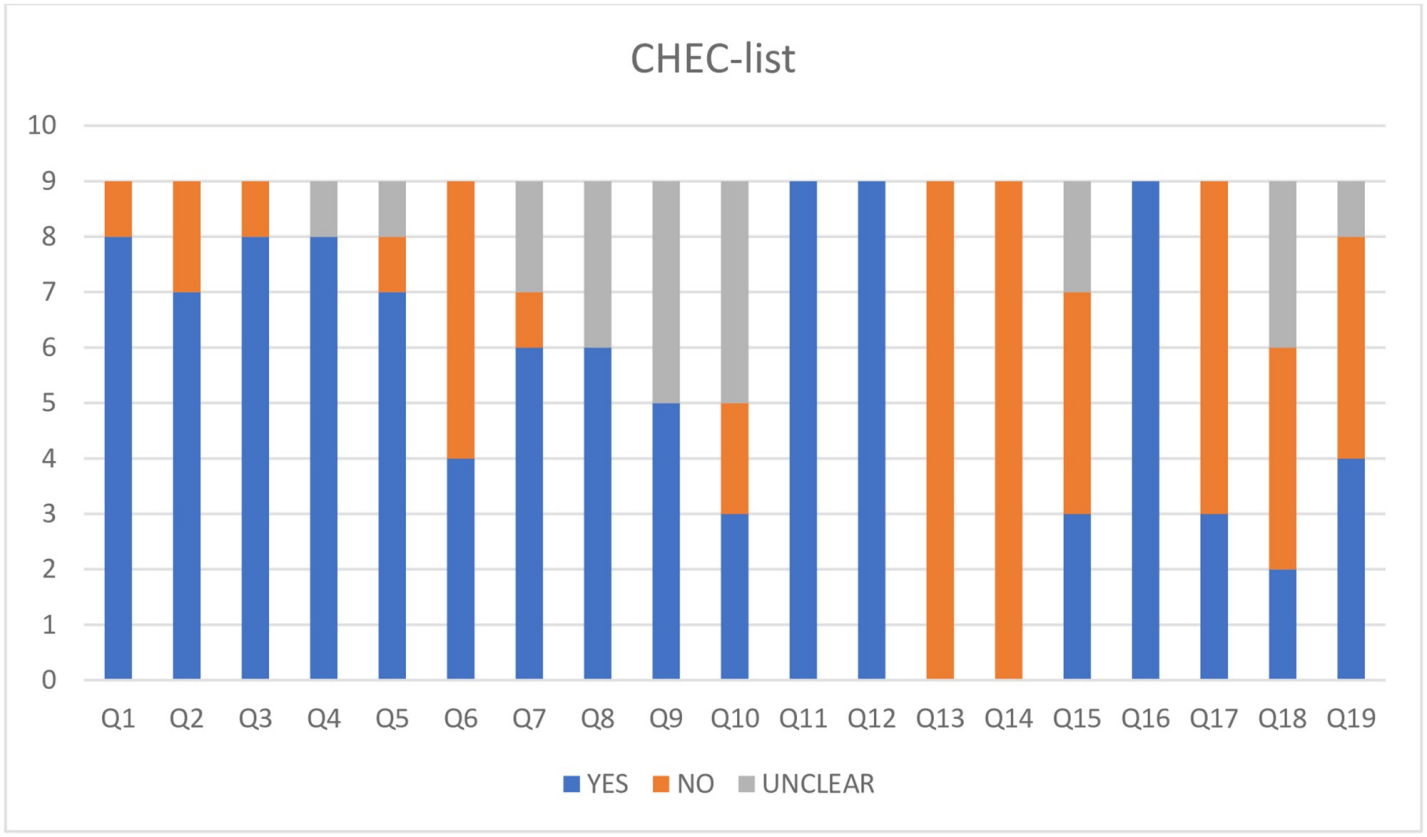
CHEClist (Evers et al.; 2005) [see S2 Table for more detail].

**Fig 3:**
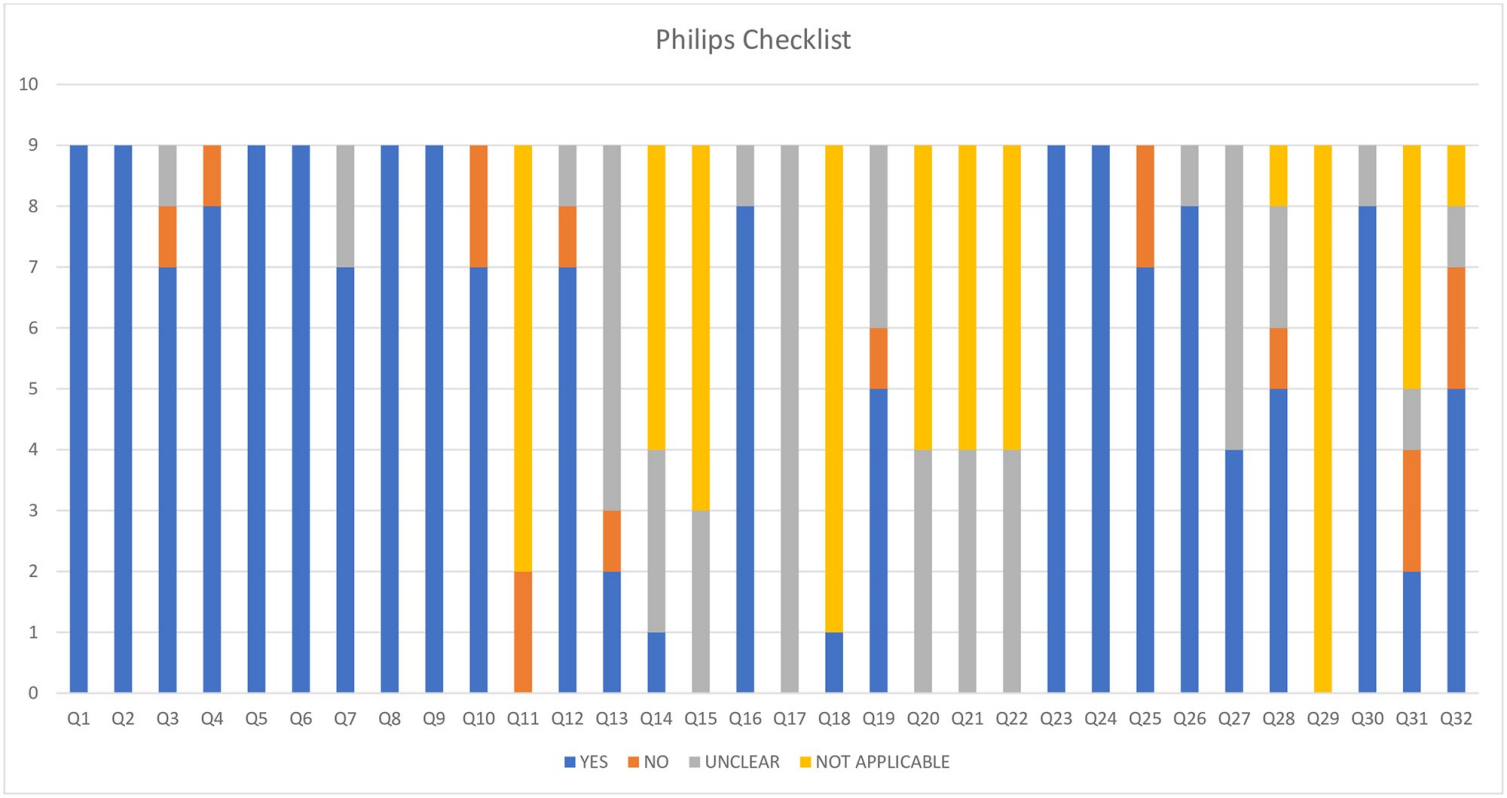
Phillips Checklist (Phillips et al.; 2005) [see S3 Table for more detail].

Overall, there were key limitations around the quality of the studies identified, particularly in relation to poor consideration of generalisability, perspective and analysis methods. Results of the application of the CHEC-list and Phillips checklist are presented in Figs 2 and 3.

**Fig 4:**
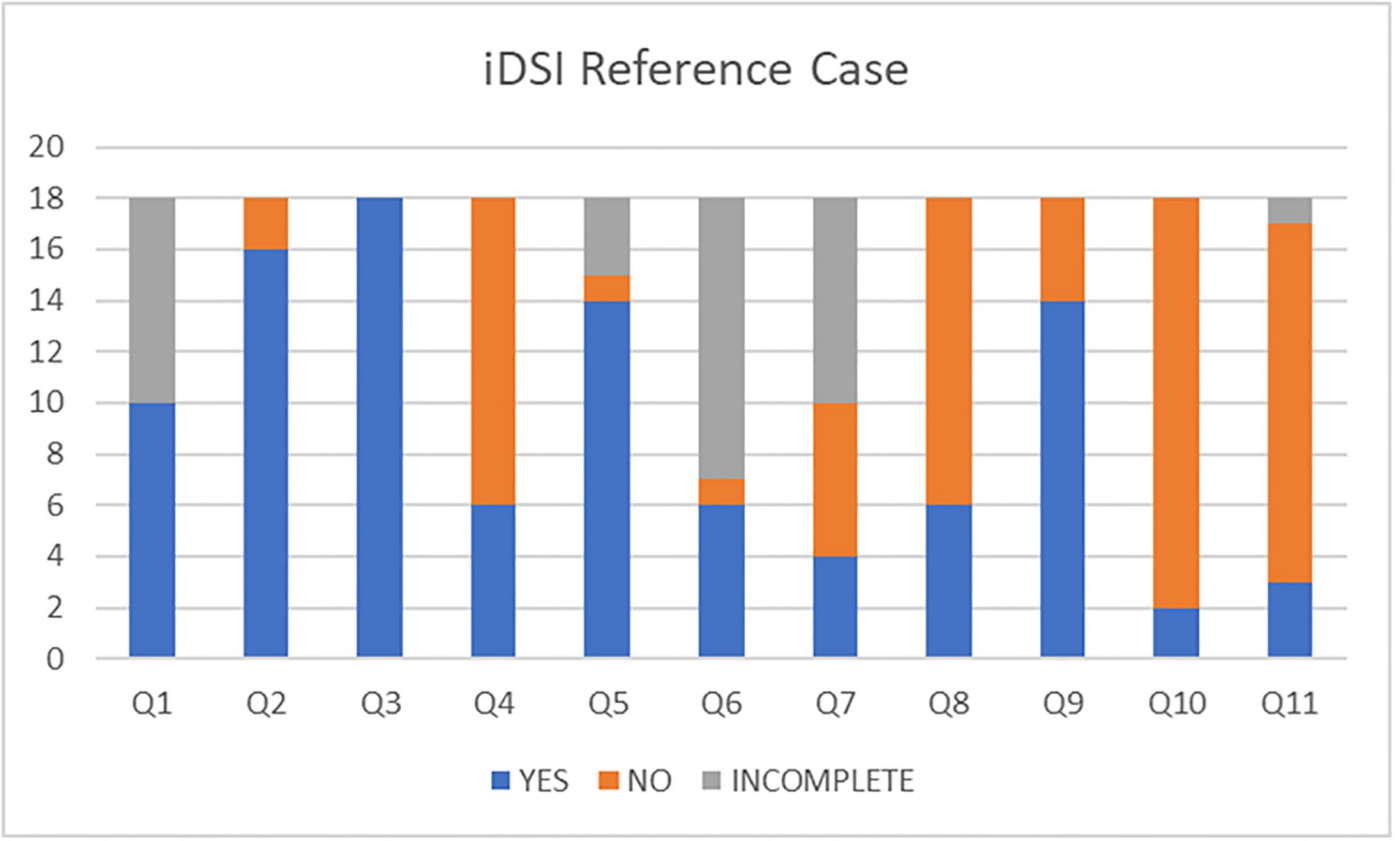
iDSI Reference Case (Wilkinson et al.,2016) [See S4 and S5 Tables for more detail].

The assessment of quality using the iDSI reference case highlighted limitations given the LMIC context of the studies (Fig 4). There were strengths around transparency and costing. However, the budget impact of the interventions was only considered by two studies. Lo et al [27] and Croce [22] concluded in their studies that cost-effective options were not always affordable. Only three studies [19,20,22] explicitly took into account equity considerations (Q11), albeit briefly (S4 and S5 Tables).

### Cost-effectiveness findings

The studies found that schistosomiasis interventions were always cost-effective compared to a “do-nothing” approach (Table 4). The studies found that a range of interventions are likely to be cost-effective for preventing, controlling, and treating schistosomiasis, compared to a comparator. For example, Yu et al [29] stated that mass chemotherapy was more cost-effective than selectively only treating confirmed positive cases (screen chemotherapy). Community based, school-based and school-aged mass drug administration (MDA) were all cost-effective dependant on the setting and local prevalence of schistosomiasis [20,23,26,28,33]. Guyatt et al. [24] and Brooker et al. [19] found that school-based MDA was cost-effective in averting childhood anaemia, which can have long-lasting effects on childhood development and learning abilities. The use of snail control was also explored by Lo et al. [32] and was found to be highly cost-effective when combined with MDA.

**Table 4:** Results and Conclusions.

No.	Author (year)	Primary Result	Results	ICER	Conclusion	Limitations acknowledged by authors	Strengths mentioned
**1**	Yu et al. (2002)[29]	Mass chemotherapy- most cost-effective. Screen chemotherapy- least cost-effective.	The costs per schistosome infection case prevented over a two-year period was RMB yuan 140.2 for Clue-group, RMB 161.2 yuan for the Mass-group and RMB 190.0 yuan for the Screen-group. The cost per 1% reduction of EPG in schistosome infection for Clue, Mass and Screen groups was RMB 169 yuan, RMB 176 yuan and RMB 145 yuan respectively. The cost per liver abnormality prevented in descending order was Clue, Screen and Mass groups. The cost per spleen abnormality prevented in descending order was: Clue, Screen and Mass groups.	-	Mass chemotherapy was the most cost-effective per case prevented and percentage reduction in the intensity of the infection. Praziquantel can significantly decrease the prevalence, and infection intensity in the endemic area studied.	1. Clinical trial data collection occurred during a short period (2 years) and was only evaluated 6 months later. This short period of intervention is less likely to capture the full effects that the interventions have produced.	-
**2**	Leslie et al. (2011)[25]	The cost-per-infection averted in adults is higher than in children.	**Direct** treatment costs: Cost/infection averted over 2-year intervention period—Adults: US$ 6.5; Children: $1.10. Combined adults and children: US$ 2.5. **Indirect** treatment costs: Cost-per-infection averted over the 2-year intervention period- Adults: US$ 4.76; Children: US$ 0.78. Combined adults and children: US$ 1.78.	-	The use of community-based delivery of MDA is of value in regions in which school enrolment is low, allowing more children to be reached.	1.The survey related to resource use in 2005 was conducted in 2006- possibility for recall bias. 2. Delay in analysing research results- possible disruption in continuity of research staff, ie changeovers.	The use of the health results of the MDA intervention as a measure of effectiveness for the study allows the direct and indirect effects created by treatment to be quantified- as done by two other previous studies.
**3**	Croce et al. (2010) [22]	The annual cost-per-infection avoided: US$ 1.33.	1.Cost/infection averted over the 9-year period: US$ 61.50 2.Cost/death averted over the 9-year period: US$ 6531 3. The Do-nothing approach and the intervention approach cost $180,000 and $737,982 over a 9-year period, respectively 4. For productivity: US$ 1 invested in the schistosomiasis control programme produced US$ 3.84 through increased productivity 5. From a Ministry of Health perspective, the CBV = 0.24.	US$ 7.02 per person treated	MDA of chemotherapy is cost-effective, but unaffordable for Cambodia. Donor funding will be needed to assist the health department in sustaining the MDA programme.	1.The Do-nothing approach costs estimation was significantly lower than the intervention. The low costs may be inaccurate as many people with schistosomiasis infection are not aware of it and do not present to health facilities. Symptoms are also quite non-specific making it easier to miss. 2. This economic evaluation did not account for the benefits that will be obtained with treatment of non-severe infections, and the improvement in schooling attendance and participation that have been documented by other studies to occur. 3.Not generalisable to African setting as endemic regions are more spread out—less concentrated.	Generalisability to other regional countries such as Laos PDR, with similar prevalence and disease.
**4**	Carabin et al. (2000b) [21]	Most cost-effective is treating those reporting blood in stool (option 3); however, 92% of infected patients would be missed. This option was therefore excluded from further analysis.	The *Kato* approach was more cost-effective than the *Symptoms* option with cost-effectiveness ratios of US$ 4.20 and US$ 12.43 per infected person treated, respectively. It was noted that the higher the prevelance of *S*. *mansoni* in the area, the smaller the cost-effectiveness ratio between these two options, however, the *Kato* approach was still always more cost-effective than the Symptoms one.	-	The use of blood in stools to diagnose *S*. *mansoni* is unsuitable for a control programme in the setting provided. The *Kato* approach is the most cost-effective approach for the setting. The cost-effective ratio of strategies will fluctuate dependant on the prevalence of infection and drug prices.	-	First study to assess Cost-effectiveness of different screening methods for treating *S*.*mansoni* in a PHC setting.
	Brooker et al. (2008) [19]	US$ 3.19 per case of anaemia averted with CE ranging from (US$1.70- US$ 9.51). Sensitivity analysis-decreasing medication price by 10% and 20% lead to cost per anaemia case averted of US$ 3.07 and US$ 2.94 respectively.	Cost-effectiveness increased with an increasing amount of children treated, and the increasing difference in the proportion of anaemia cases averted by the intervention. Cost-effectiveness was decreased by an increasing cost-per-individual treated. Significant variation existed amongst the cost of individuals treated in the different districts.	-	Economies of scale exist for STH- schistosomiasis control programme. Intra-country variation in cost and cost-effectiveness exists and needs to be taken into consideration when implementing policies based on study results. Repeating study with larger cohort may be of value.	1) External contributors may exist that contributed to the decreased number of anaemia cases. 2) Effectiveness metric used is not comprehensive and is not easily comparable.	Anaemia cases averted is an effectiveness metric that can be easily assessed.
**6.**	Zhou et al. (2005) [35]	1a.RMB Yuan 12.48 per case detected 2.US$ 6.20 gained for every US$ 1 spent. Net BCR = 6.20	1b. RMB Yuan 41.86 per individual in project 1c. RMB Yuan 2176.72 for case detection to reduce 1% human infection rate per 100 individuals. 1d.RMB Yuan 773.42 to reduce 1% human infection rate per 100 individuals in project 1e.RMB Yuan 16 289.10 to reduce 1% bovine infection rate per 100 cattle.	-	Areas of increased prevalence had an increased benefit-per-cost invested in project. The project has reduced prevalence of *S*. *japonicum* in China.	-	-
**7**	Yu et al. (2013) [34]	**Unit cost of reducing human S. japonicum infection by 1%** 1.Comprehensive programme (Case Village) Zhangjia YR 2- US$ 4100, year 3: US$2,500, year 4: US$ 80 Jianwu—year 2: US$ 400, year 3: US$ 4,300, year 4:US$ 80 2. Standard programme Koutou–year 2: US$ 500, year 3: US$ 200, year 4: US$ 200, Xiajia—year 2: US$ 200, year 3: US$ 400, year 4: US$ 40	**Unit cost of reducing cattle S. japonicum infection by 1%** 1.Comprehensive programme Zhangjia YR 2- US$ 120,600, YR 3- US$ 1400, YR 4- NOT STATED. Jianwu—YR 2- US$ 429,400, YR 3- US$ 2900, YR 4 US$ 100. 2. Standard programme Koutou YR 2- US$ 150, YR 3- US$ 130 YR 4- US$ 600. Xiajia YR 2- US$ 400, YR 3 US$ 300, YR 4- US$ 300. Prevalence decreased from baseline in both case villages, but one control village had an increase in baseline: Zhangjia (case) 11.3% to 1.6%, Jianwu 6.7% to 0.6%. Koutou 6.5% to 3.2%, Xiajia 8% to 13%.	-	Comprehensive programme is cost-effective at reducing *S*. *japonicum* infection.	-	-
**8**	Guo et al. (2005) [30]	The intervention of passive chemotherapy and health education was more cost-effective than the standard MDA.	Case group: 1998/1999 RMB yuan 18.2 per infected person who received treatment 1999/2000 RMB yuan 24.2 per infected person who received treatment Control group: 1998/1999 RMB yuan 44.6 per infected person who received treatment 1999/2000 RMB yuan 77.1 per infected person who received treatment	-	In areas that have had a successful control programme running for some time, passive chemotherapy and health education is more cost-effective than mass chemotherapy for continuation of results of the initial control programme.	There is a risk of missing asymptomatic infection.	High acceptance of the intervention amongst locals.
**9.**	Guyatt et al. (2001) [24]	Decreased prevalence of anaemia 25%. Mod-severe cases of anaemia decreased by 50%. `	US$ 7.23 to US$ 145.71 per-anaemia case averted dependent on haemoglobin threshold used. Threshold range (70-120g/l). Non-linear results. Cost significantly hight at low Hb thresholds, but stable at thresholds above 100g/l. Combining the decreased prevalence and costs and using a school-based delivery and administration of medication, the cost per anaemia case averted could be US$ 7.43 per anaemia case averted.	-	School-based deworming programmes can cost-effectively decrease the severity and amount of anaemia cases in SAC.	The outcome measure does not quantify other benefits that occur as a result of deworming.	-
**10**	Collyer et al. (2019) [36]	Based on cost alone, the WTP for a vaccination programme over MDA is US$ 3.70/vaccination course.	After 10 year period: transmission not interrupted 20 years: heavy intensity infections are eliminated 30 years: interruption of transmission Community-based treatment leads to larger and quicker reductions in the prevalence of infection. If a vaccine has a duration of efficacy of under 20 years, immunisation of SAC is the most cost-effective vaccine strategy per heavy intensity infections averted. MDA becomes more cost-effective as coverage increases.	-	CE dependant on transmission dynamics, vaccine efficacy duration and the cost of the vaccine.	Unclear	-
**11**	Lo et al. (2016) [27]	Schistosomiasis PC was highly CE for SAC at a prevalence above 5%, and community-wide treatment at 15%. STH PC was highly CE for SAC at a prevalence above 20% and community-wide treatment at 60%.	Annual SBT MDA is cost-effective for STH at 20% prevalence and schistosomiasis at 5% prevalence in SAC. Co-endemic areas required significantly lower prevalence rates for CE of PC- STH only requiring a prevalence of 1–5%. Prevalence thresholds for annual PC to SAC was robust.	ICERs Ranging from US$ 160 to US$ 1077 per DALY averted.	1. Annual MDA for schistosomiasis and STH is estimated to be cost-effective at a prevalence lower than what WHO currently recommends. 2. 21.3% of SSA can benefit from an integrated programme where both diseases are co-endemic. 3. Cost-effective treatments may still be unaffordable especially in low-income countries.	Very generalised model. Cost-effectiveness threshold may need to be adjusted for individual countries. Long-term effects of helminths may be underestimated based on effectiveness metric used.	Generalisability
**12**	Lo et al. (2015) [26]	CWT more highly CE than only SAC treatment Cost-effectiveness is robust.	SAC and preschool-aged children, and adults alone options dominated by Community-wide treatment The prevalence of helminth infections in adults was greater than that in school-aged children (SAC) in all 4-communities. To get towards elimination most helminth species required ≥ 75% coverage for a sustained period of ≥5 years and community-wide treatment. Coverage ranging from 5–100% produced results of US$ 149–277 per-DALY averted Integrated bi-annual treatment highly cost-effective. Co-administration of MDA increases benefits by decreasing incremental costs-> increased cost-effectiveness at a lower prevalence.	ICER US$ 118 per-DALY averted SAC ICER US$ 167 per-DALY averted–Community-wide treatment. Community-wide treatment vs WHO guidelines- ICER US$ 127 per-DALY averted. PSA: mean ICER US$ 219 per-DALY averted.	1) CWT STH and schistosomiasis is highly cost-effective, with results showing robustness	1. Contributions of animal reservoirs, migration, ‘super-spreaders’ were excluded. 2.The 15-year period modelled- treatment coverage assumed to be constant throughout. 3. Disability weights- used one disability weight for schistosomiasis, and varying disability weights in literature. 4.Treatment costs were not divided into initial capital costs and variable costs.	-
**13**	De Neve et al. (2018) [23]	US$125 per DALY averted (ICER) Benefit Cost Ratio (BCR) = 13 (5–31)	Large geographical variation in the costs of treating NTD in Madagascar. Integrated NTD programmes are more cost-effective than individual programmes Alternative scenario using a value of US$ 250 per DALY averted, BCR = 7 (1–19)	US$ 125 per DALY averted	Regional programmes should be considered	1.Double counting risk on benefits 2. Reliance on limited available evidence 3. inability to capture all benefits 4. Decreased CE of NTD programme over time not considered 5.Focus on prevalence as opposed to individuals with heavy infections- which account for most morbidity 6.Static model 7. Drug of choice affects estimates 8.No capture of no of poverty cases averted	Includes non-health benefits, includes social and educational benefits giving policymakers more information on effect
**14**	Carabin et al.(2000a) [20]	Treating both school-aged and school-enrolled children can be as cost-effective or more cost-effective than just treating school-enrolled children.	*S*. *mansoni* and *S*.*haematobium* results for each strategy and each outcome reported-tabulated	-	Treatment on non-enrolled children can improve effectiveness with minimal changes in cost-effectiveness	1.Individuals are assumed to stay in the same sex and school enrolment group throughout. 2.Groups are assessed, not individuals 3. Only prevalence data was received (no data on intensity)	-
**15.**	Ndeffo-Mbah et al. (2013a) [33]	**1st scenario- MDA reduces HIV transmission**- 30% efficacy MDA annual dose–US$ 259.31/HIV case-averted over intervention period. 70% efficacy US$ 51.68/HIV case averted. **2**^**nd**^ **scenario-MDA reduces FGS**– 30% efficacy US$ 131.94/HIV case averted. 70% efficacy US$ 56.50 / HIV case averted.	**1**^**st**^ **scenario- MDA reduces HIV transmission** 30% efficacy MDA annual dose–Net saving of US$ 15.8 million in medical care cases averted. 70% efficacy net saving US$ 101.4 mill in medical care cases averted. **2**^**nd**^ **scenario-MDA reduces FGS** 30% efficacy-Net savings US$ 36.4 million in medical cases averted 70% efficacy -Net savings of US$ 92.3 million in medical cases averted		Increasing MDA for schistosomiasis in endemic areas of SSA will be a potentially cost-effective intervention for HIV prevention	1. Cost-effective results are conservative as the model does not fully explain the age-structure dynamics of *S*. *haematobium* 2. The model does not account for the potential age acquired natural partial immunity to FGS that may occur 3. Epidemiological evidence is from cross-sectional studies	-
**16**	Ndeffo-Mbah et al. (2013b) [28]	It was cost-effective to add WASH to school-based treatment MDA even when WASH strategies had efficacy as low as 30%- $875 per individual over 20 year period. WASH 90% CE if efficacy between 10–70%. Longer duration of intervention significantly increases its cost-effectiveness	PSA: Cost-effectiveness of community-based treatment was more sensitive to the cost of WASH, as opposed to the efficacy. Longer duration of community-based treatment increased its cost-effectiveness	-	Integration of WASH and chemotherapy may have many benefits beyond HIV prevention—public health benefits	-	-
**17.**	Lo et al. (2018) [32]	**PREVALENCE REDUCTION:** MDA SAC> snail intervention alone. Community-wide MDA> SAC MDA or snail intervention. **PREVALENCE AND INTENSITY OF INFECTION REDUCTION**: Combination snail intervention and MDA greatest reduction, but decreased marginal effectiveness High burden areas with snail control had a lower ICER	**BASELINE**: Low burden 172 DALYS over 10 year. High burden 550 DALYs. **LOW BURDEN**: bi-annual SBT MDA with bi-annual snail control- highly cost-effective ($904) and annual CWT with bi-annual snail control US$ 1531/DALY averted. **HIGH BURDEN**: annual CWT with bi-annual snail control- US$ 588/DALY averted. Bi-annual CWT with bi-annual snail control US$ 1213/DALY averted.	See Results column on left	Snail control with MDA is highly cost-effective for schistosomiasis control. The optimal strategy depends on the prevalence of the community	1. Super-spreaders not factored in 2. Seasonality not created 3. Environmental and ecological consequences of using chemical molluscicide- not factored in	-
**18**	Kirigia (1998) [31]	All interventions except the vented pit latrines are more cost-effective than the status quo The most cost-effective strategy was selective population chemotherapy with praziquantel	-	Disaggregated—Foreign expert and local expert estimates	The use of estimates by experts is necessary where evidence is lacking, as policy decisions still need to be made	Unable to generalise due to the use of non-validated methods	-

- = Not described, Co-endemic areas = Areas with prevalence of both STH and schistosomiasis

BCR–Benefit cost ratio, CBV–Cost benefit value, CE—Cost-effective, CWT—Community-wide treatment, DALY—Disability-adjusted life year, EPG -Eggs per gram of faeces, HIV—Human immunodeficiency virus, ICER—Incremental cost-effectiveness ratio, MDA—Mass drug administration, NTD—Neglected Tropical Disease, PC—Preventative chemotherapy, PHC—Primary health care, PSA—Probabilistic sensitivity analysis, SAC—School-aged children, SBT- school-based treatment, SSA—Sub-Sahara African, STH—Soil-transmitted homophiles, WASH—Water, Sanitation and Hygiene, WTP—Willingness-to-pay.

There existed some conflicting results. Yu et al. [29] found that MDA was more cost-effective than selective chemotherapy, whereas Guo et al. [30] and Kirigia [31] reported the converse. Lo et al. [26] further identified community-wide PC treatment (CWT) of schistosomiasis and soil-transmitted helminths (STH) was highly cost-effective, whilst Lo et al. [27] found only SAC selective chemotherapy to be robust through sensitivity analysis. Lo et al. [27] additionally stated that the cost-effectiveness of interventions was dependent on the prevalence of the condition (Table 4).

Only six studies reported their incremental cost-effectiveness ratios (ICERs) [22,23,26,27,31,32] (Table 4). Three of these studies reported ICERs that were inclusive of cost-effectiveness measures for other NTDs [23,26,27]. With different effectiveness measures being used, the ICER ranged from US$ 7.02 per person treated in a Cambodian national MDA programme against *S*. *mekongi* to as high as US$ 1,531 per DALY averted through an integrated snail-and-chemotherapy intervention against *S*. *haematobium* in low-burden areas in Kenya [22,32]. One study considered a schistosomiasis vaccination which had a hypothetical basis, therefore, no concrete decision was able to be made about its cost-effectiveness [36]. Ndeffo-Mbah et al. [33] found that having a schistosomiasis PC programme with the addition of water, sanitation and hygiene (WASH) interventions or snail control (mollusciciding) was potentially cost-effective in HIV prevention, with additional public health benefits (Table 4).

Only two papers touched on the affordability of the interventions [22,23]. Both studies concluded that although their findings showed that MDA was cost-effective in their respective settings, the programmes were still unaffordable for the context.

Most of the analysed papers did not state that any cost-effectiveness threshold (CET) was used to present their findings. Amongst the seven papers that applied a threshold, different thresholds were utilised. Yu et al. [29] used a weighting system to rank the outcomes in ascending order of importance. Collyer et al. [36] used a benchmarking technique to calculate a critical vaccination cost. They deemed the cost-effective threshold as being a vaccination cost that was equal to or less than that of MDA. Lo et al. [26,27,32] and Ndeffo-Mbah et al. [33] used their respective countries’ and region’s GDP per-capita amounts, as their cost-effective thresholds.

Three papers conducted a CBA alongside a CEA [22,23,35] and estimated a range of benefit cost ratios (BCRs). De Neve et al. [23] explored a CBA in addition to their CEA and converted the benefits of NTD control (such as DALYs and educational gains) into monetary units and divided this value by the total cost of the NTD control programme investment. The BCR of the entire control programme was estimated to be 13 (5–31), with an alternative scenario yielding a BCR of 7 (1–19) (Table 4). Zhou et al. [35] also conducted a CBA and the net BCR of their national schistosomiasis control programme was 6.20 (Table 4). The authors noted that limited literature on CBA of schistosomiasis control programmes existed at the time of their economic evaluation [35]. Croce et al. [22] explored both a societal and Ministry of Health (MoH) perspective. From a societal productivity perspective, the programme was noted to be economically beneficial, yielding US$ 3.84 per US$ 1 spent (Table 4), whereas the narrower MoH perspective yielded a cost-benefit value (CBV) of 0.24 [22].

## Discussion

### Principal findings

Very few studies were identified which considered the costs and outcomes for interventions to prevent, control and eliminate schistosomiasis. The evidence, which does exist, suggests that a range of interventions are likely to be cost-effective compared to a ‘do nothing’ approach. However, the review identified some methodological limitations in the existing literature and highlighted a need for greater standardisation of economic evaluations in this area.

A societal perspective was not widely adopted, which may lead to the benefits of the interventions being underestimated. NTDs often have a significant effect on the long-term productivity of the affected individuals, by causing chronic disease that impairs not only an individual’s ability to work physically but also their mental wellbeing [2]. This decreased productivity can translate to a loss of income which would affect the worker and their families. For NTDs, where possible, it is important to adopt a societal perspective in an economic evaluation to ensure that the impacts on employment and families are factored into decision making. Furthermore, the effects on productivity may be pivotal in decision-making processes, such as whether to fund a school-based versus a community-based control programme.

The variation in the time-horizon used in the studies was vast, hampering comparison of studies. There was no lifetime-horizon employed, which would be of value in a disease of possible lifetime duration. The reasons behind the time-horizons adopted were not fully explained in many studies.

Policy makers and programme funders need to scrutinise the epidemiology and prevalence rates used in economic evaluations and assess if they align with their region-specific prevalence rates, as this will affect the extent of the EEs generalisability. De Neve et al. [23] and Brooker at al. [19] highlighted that regional variation existed in their respective countries within a single schistosomiasis intervention programme. The targeting of predominately SAC children for MDA is appropriate, as children are known to be at an increased risk of infection due to underdeveloped immunity, and tend to harbour heavy infection [2,26,28] and the disease can produce long term adverse effect on development in childhood [2]. Furthermore, the prevalence of schistomiasis in SAC in a region determines how frequent MDA will be administered in the respective region [2].

The disability weights allocated to DALYs in schistosomiasis have been heavily criticised for underestimating the effect of the disease [38–40]. Whilst significant improvement has been made in the weighting of schistosomiasis DALYS since the 1990 Global Burden of Disease report, many authors still believe that currently DALYs do not fully capture the effects of schistosomiasis [38–40].

The reasons for this have been attributed to exclusion of serious disease complications and inadequate incorporation of new disease-related information into weightings, and limited understanding of the disease complexity [11,38–40]. Schistosomiasis is a complex disease, and the effect on morbidity of infection diagnosis in childhood and adulthood in endemic areas differs. There are certain complications that when diagnosed later in adulthood cannot be rectified by treating the disease and are independent of the levels of intensity of disease at the time of diagnosis [40]. There is evidence to suggest that reducing the intensity of infection can create a reduction in schistosomiasis related morbidity [41]. Heavy and light infection with schistosomiasis have been assigned different disability weightings; however King et al. [42] argues that factors such as inflammation mediated by the immune system in schistosomiasis is found to not be attributable to intensity of infection. This creates uncertainty around weightings given to light infections, as their effects on morbidity have been found to be underestimated. Diagnosis of this may be missed leading to underestimation of their impact [40].

Turner et al [40] recommend that urgent attention should be given to improve the estimates of DALYs. They propose that a framework is needed that takes into consideration: differentiation of the reversible and non-reversible complications of disease and which can distinguish between levels of infection in different age groups and Schistosoma species [11]. It is therefore important that future economic evaluations are transparent about how DALYs have been calculated and highlight associated limitations. Funders and health policy makers need to be aware of these issues, and their effects on future DALY calculations, and transmission models for schistosomiasis.

Four studies [26–28,32] found their interventions to be cost-effective when utilising the WHO GDP based CETs (Table 3). The WHO has in recent years encouraged LMICs to adopt local CETs, that can take their available financial resources into consideration [43,44]. Bertram et al. [43] acknowledged that whilst the WHO GDP based CET may be helpful in guiding assessments, they are not to be used in isolation by policy makers for deciding on health intervention funding. Kazibwe et al. [45] conducted a review of CEAs using a cost-per-DALY metric in LMICs between 2015–2020 and found that over 80% of papers reviewed used WHO GDP values as a CET, with some studies forfeiting local thresholds to use a GDP based CET. The WHO has acknowledged that the GDP based thresholds neglect the affordability and practicality of implementation of CETs. The use of generic CETs may lead to funders and health decision makers funding programmes that are unsustainable in their context, leading to inefficient allocation of resources [43]. This highlights the importance of including criteria to assess affordability within economic evaluation checklists, to ensure optimal allocation of healthcare resources.

The iDSI reference case highlighted important considerations for economic evaluations in this context: affordability and equity. Unfortunately, the affordability of the intervention for the context-specific health budget was only mentioned in two papers [22, 27] and only three studies considered equity issues [21,23,25]. The importance of highlighting affordability and equity considerations in NTDs cannot be overemphasised. NTDs tend to affect the most vulnerable populations globally, and without considering equity and affordability, studies are unlikely to address key concerns for decision makers.

### Comparison to the existing literature

Very few systematic reviews relating to economic evaluations of interventions for schistosomiasis or NTDs more generally have been undertaken. Turner et al. [11] conducted a systematic review of the economic evidence relating to human schistosomiasis and identified a higher number of studies than the current review, as they included studies that were not formal economic evaluations [11]. The authors highlighted issues relating to the types of models and outcome measures used. The study focussed more on the results of the included studies than on the methods used and did not employ a quality assessment tool.

Garcia et al. [10] conducted a systematic review of peer-reviewed papers on economic evaluations of interventions against STH and schistosomiasis between 1990 and 2012 and used the Consolidated Health Economic Evaluation Reporting Standards (CHEERS) checklist to appraise quality. They found that the papers performed well and would be beneficial in policymaking. The differences in the results between this current review and those of Garcia et al. [10] may be attributable to the different quality appraisal methods used with the CHEERS checklist more focussed on reporting rather than methodological quality.

### Strengths and weaknesses of the study

The strengths of the review included that a broad and comprehensive search was conducted using general and inclusive terms, and that a systematic categorisation strategy was employed. This review analysed the methodological quality of economic evaluations using the CHEC-list, the Philips checklist and the iDSI checklist. The use of the iDSI checklist is novel in this context and allows consideration of factors particularly crucial in an LMIC context.

The studies were limited to papers published in the English language—which means there exists the risk of accidental exclusion of valuable untranslated papers. There was considerable variation in the methods and outcome measures used in the papers which made comparison of studies difficult.

The starting year for inclusion of papers was 1998 based on the WHO-CHOICE initiation. In reality, however, studies (particularly trial-based) would have taken some time to adapt to the new recommendations. The iDSI (previously known as the Gates reference case) was only established in the year 2014 [46], however even the more recent studies did not fully adhere to all criteria.

### Meaning of study: Possible implications and mechanisms for policymakers

Schistosomiasis interventions were collectively found to be cost-effective, but the quantity and quality of studies was generally limited, with a need for greater standardisation. Economic evidence is particularly important for NTDs, which by definition have tended to be neglected in resource allocation decision-making. The identified studies used a range of outcome measures, which hinders the comparison of cost-effectiveness. Furthermore, the outcomes considered are generally not broad enough to cover all the effects of schistosomiasis interventions, with many focussing on clinical outcomes alone. Based on the chronicity and long-standing effects of untreated or missed disease, a lifetime-horizon is likely to be appropriate for interventions in this area. However, if this is not possible, consensus regarding the time-horizons appropriate for different types of intervention is needed. Consideration of equity and affordability is likely to be needed for decision-making in this context.

The use of CBA has some strengths as the use of monetary benefits allows comparison across a range of different sectors and disease areas. However, some limitations of this approach in relation to NTDs have been highlighted. Zhou et al. [35] noted that the use of CBA creates the complicated situation of putting a monetary value on the welfare and wellbeing of people in certain regions. Furthermore, schistosomiasis and other NTDs mostly affect communities who are already unfairly disadvantaged, and hence there is a risk that focusing on monetary benefits will contribute to the continued neglect of control programmes, and perpetuate health inequities. Hence, caution and further research is needed.

Only two economic evaluations included WASH interventions [28,34]. Access to water and sanitation is considered a human right, and is enshrined in the sixth SDG [4,47]. The availability of WASH in communities has been proven to improve child and maternal health, as well as reduce and control infectious diseases including diarrhoeal diseases and NTDs. Diarrhoeal diseases are a significant source of mortality in children under five, particularly in LMICs [47]. WASH is known to be a critical component of schistosomiasis control and helps provide a more sustainable effect on reduction of schistosomiasis transmission [48]. The cost and increased amount of commitment potentially required for implementing WASH interventions may have created a barrier to WASH inclusion [49]. The cost-effectiveness of WASH interventions thus requires further investigation and analysis.

The combination of schistosomiasis and STH programmes has been shown to be cost-effective in the studies looking at combined programmes. The common co-existence of STH in endemic schistosomiasis areas and the regular combination of STH and schistosomiasis statistics [2,50] makes this a feasible option that needs more exploration in future economic evaluations. Policy makers will however have to reassess the cost-effectiveness of these thresholds within their respective local context, and health resource availability. Future EEs should move away from reliance on WHO GDP-based CETs.

The effects of migration, improvement in WASH, and climate change will all contribute to the changing epidemiology of schistosomiasis in the regions [51]. This will mean that policy makers and programme funders will need to ensure that up to date region specific prevalence estimates are incorporated into calculations. Surveillance systems need to be strengthened and maintained in endemic areas. With WHO advocating for disease control and an eventual break in transmission and then elimination, there needs to be a shift from analysing the cost-effectiveness of schistosomiasis control interventions to a future focus on the cost-effectiveness of interventions to eliminate schistosomiasis.

## Conclusion

This systematic review has demonstrated that the current economic literature around schistosomiasis interventions has limitations, particularly in relation to perspective, time-horizon and consideration of equity and budget concerns. There is a need for greater standardisation of the methodology used in the evaluation of interventions to target NTDs, particularly schistosomiasis. Improved standardisation of studies would allow greater transparency and generalisability of the economic evidence, which is important for decision-makers in allocating funding for these programmes.

Controversy still exists around the use of DALYs in schistosomiasis studies. With the use of DALYs still being considered a methodological standard in LMIC economic evaluations, further research is required to refine the calculation of DALYs or create a more precise outcome measure.

Integration of related or associated diseases should be considered in future studies, as has been undertaken for HIV-female genital schistosomiasis (HIV-FGS) and STH-schistosomiasis economic evaluations. This is because diseases do not exist in isolation and often have associations. By highlighting these disease interactions in dynamic transmission models, there might be increased evidence for decision-makers to help optimise policy in this area. Health-economics is still a growing field for NTDs, and additional support is needed to increase the quantity and improve the quality of economic evidence in this important area of human health.

## Supporting information

S1 PRISMA Checklist PRISMA 2020 Evaluation. (PDF)

S2 PRISMA Checklist PRISMA 2020 Abstract Evaluation. (PDF)

S1 Glossary Glossary of Essential Economic Terms. (PDF)

S1 Table Key Assumptions of model-based economic evaluations. (PDF)

S2 Table CHEClist criteria. (PDF)

S3 Table Philips checklist criteria. (PDF)

S4 Table iDSI reference case—Trial-based economic evaluations. (PDF)

S5 Table iDSI reference case—Decision-analytical models. (PDF)

S6 Table EMBASE search strategy: 1 January 1998–17 July 2020. (PDF)

S7 Table Web of Science search strategy: 1 January 1998–17 July 2020. (PDF)

S8 Table MEDLINE and MEDLINE In-process and non-indexed citations search strategy:1 January 1998–17 July 2020. (PDF)

S9 Table EconLit search strategy: 1 January 1998–17 July 2020. (PDF)

S10 Table Funding sources declared by authors. (PDF)

S11 Table Hand search exclusion tables. (PDF)
