# Supplementary material for: A systematic review and quality appraisal of the economic evaluations of schistosomiasis interventions
Source: PLoS Negl Trop Dis. 2022 Oct 12;16(10):e0010822. doi: 10.1371/journal.pntd.0010822 (PMC9591071; doi:10.1371/journal.pntd.0010822)
Supplement: S1 Glossary — (PDF) [file pntd.0010822.s003.pdf]

S1 Glossary

Essential Economic Terms

Complete Economic Evaluation:
A study involving the comparison of costs and outcomes of two or more alternative interventions.

Partial Economic Evaluation:
A study involving the comparison of either/or a combination of costs and outcomes without fulfilling the criteria of a complete economic evaluation.

Cost-Effective analysis (CEA)
CEA refers to an economic evaluation in which the outcomes of an intervention are expressed in non-monetary units.

Cost-Benefit analysis (CBA)
CBA consists of economic evaluations that use a monetary unit to measure and compare both costs and effects.

Cost-Utility Analysis (CUA)
CUA is a type of CEA that use a generic unit to quantify health outcomes such as DALY, QALY or health-years equivalent amongst others.

Static decision model
Static models are the simplest and most commonly used decision models. They are usually simple Markov models or decision trees. Static models assume that the probability of disease is constant, which makes them less suitable for infectious diseases.

Dynamic decision model
Dynamic models take into account the prevalence and transmission cycle of the disease. They can capture the indirect effects such as that of herd immunity that occurs in mass vaccination campaigns when an adequate number of individuals receive preventative vaccines. They can also be used to estimate the cost-effectiveness of elimination of diseases with particular interventions. They are most suitable for infectious disease
