# Supplementary material for: A systematic review and quality appraisal of the economic evaluations of schistosomiasis interventions
Source: PLoS Negl Trop Dis. 2022 Oct 12;16(10):e0010822. doi: 10.1371/journal.pntd.0010822 (PMC9591071; doi:10.1371/journal.pntd.0010822)
Supplement: S1 Table — (PDF) [file pntd.0010822.s004.pdf]

S1. Table Key assumptions of model-based economic evaluations

No.	Author (year)	Key assumptions
**1**	Collyer et al. (2019) [1]	1. Immunisation has an immediate effect<br>2. Immunisation will decrease the ability for schistosomes to breed in human hosts, as well as the reproductive ability of the female worms<br>3. The vaccine does not have a treatment effect on schistosomes already present prior to immunisation<br>4. Vaccine decreases the breeding of new worms, and the reproductive ability of female worms both by 90%<br>5) Assumption in epidemiological model that the population area has poor sanitation
**2**	Lo et al. (2016) [2]	1. Negative binomial relationship between prevalence and the mean intensity of infection exists (as per published epidemiology studies)<br>2. treatment modelled as an immediate reduction in intensity of infection
**3**	Lo et al. (2015) [3]	1. Negative binomial relationship between prevalence and the mean intensity of infection exists<br>2. Individuals are susceptible to be re-infected by parasites immediately after treatment<br>3. Treatment is an immediate reduction in average parasite burden-<br>4. SAC and pre-school children are responsible for two times the relative amount of parasite eggs found in the environment when compared to adults<br>5. Treatment coverage was 75%- in line with WHO goals<br>6. Medication not donated<br>7.Heterogeneity assumed to be constant in the population<br>8.Coverage of treatment in population was considered random
**4**	De Neve et al. (2018) [4]	1. Out of pocket costs - 41% of total cost<br>2.Infections averted from each intervention are independent of other diseases
**5**	Carabin et al. (2000a) [5]	1. Age distribution assumed equal for both males and females<br>2. Treatment decreases worm burden immediately<br>3.Coverage at school assumed to be 85%
**6**	Ndeffo-Mbah et al. (2013a) [6]	1. Patients have no natural recovery from FGS.<br>2. FGS infection lead to an increase in HIV transmission by a factor that was derived from epidemiological data on HIV-FGS co-infection.<br>3. FGS is primarily acquired in childhood.<br>4. Girls treated with praziquantel during SAC (5-14 yrs) were less likely to acquire FGS.<br>5. Average age of HIV acquisition amongst Zimbabweans is 25<br>6. Onset of sexual activity is 15 years
**7**	Ndeffo-Mbah et al. (2013b) [7]	1. Girls treated with praziquantel were less likely to acquire FGS compared to untreated girls<br>2. ARV coverage 37% in SSA<br>3.Average age of HIV acquisition is 25yrs<br>4. At 25 years, if patient acquires HIV, then life expectancy 40
**8**	Lo et al. (2018) [8]	1.Effect of treatment immediate<br>2.'Perfect mixing'<br>3.MDA not donated
**9**	Kirigia (1998) [9]	1.Distribution of health states<br>2.Probability of being in a particular health state depends on the action taken at a community level<br>3.The Delphi panel experts held prior beliefs about the local population health states, and specific outcomes being experienced by patients<br>4. Health states listed are comprehensive and mutually exclusive<br>5. Kenyan life expectancy-57 years<br>6. The rate of Kenyan government bond= social opportunity cost of capital

References:

1. Collyer BS, Turner HC, Hollingsworth TD, Keeling MJ. Vaccination or mass drug administration against schistosomiasis: a hypothetical cost-effectiveness modelling comparison. Parasites & Vectors. 2019;12(1).
2. Lo NC, Lai YS, Karagiannis-Voules DA, Bogoch, II, Coulibaly JT, Bendavid E, et al. Assessment of global guidelines for preventive chemotherapy against schistosomiasis and soil-transmitted helminthiasis: a cost-effectiveness modelling study. Lancet Infect. Dis. 2016;16(9):1065-75.
3. Lo NC, Bogoch II, Blackburn BG, Raso G, N'Goran EK, Coulibaly JT, et al. Comparison of community-wide, integrated mass drug administration strategies for schistosomiasis and soil-transmitted helminthiasis: A cost-effectiveness modelling study. Lancet Glob. Health. 2015;3(10):e629-e38.
4. De Neve JW, Andriantavison RL, Croke K, Krisam J, Rajoela VH, Rakotoarivony RA, et al. Health, financial, and education gains of investing in preventive chemotherapy for schistosomiasis, soil-transmitted helminthiases, and lymphatic filariasis in Madagascar: A modeling study. PLoS Negl Trop Dis. 2018;12(12).
5. Carabin H, Chan MS, Guyatt HL. A population dynamic approach to evaluating the impact of school attendance on the unit cost and effectiveness of school-based schistosomiasis chemotherapy programmes. Parasitology. 2000;121:171-83.
6. Ndeffo Mbah ML, Poolman EM, Atkins KE, Orenstein EW, Meyers LA, Townsend JP, et al. Potential Cost-Effectiveness of Schistosomiasis Treatment for Reducing HIV Transmission in Africa - The Case of Zimbabwean Women. PLoS Negl. Trop. Dis. 2013;7(8).
7. Ndeffo Mbah ML, Kjetland EF, Atkins KE, Poolman EM, Orenstein EW, Meyers LA, et al. Cost-effectiveness of a community-based intervention for reducing the transmission of Schistosoma haematobium and HIV in Africa. Proc. Natl. Acad. Sci. U.S.A. 2013;110(19):7952-7.
8. Lo NC, Gurarie D, Yoon N, Coulibaly JT, Bendavid E, Andrews JR, et al. Impact and cost-effectiveness of snail control to achieve disease control targets for schistosomiasis. Proc. Natl. Acad. Sci. U.S.A. 2018;115(4):E584-E91
9. Kirigia JM. Cost-Utility Analysis of Schistosomiasis Intervention Strategies in Kenya. Environ Dev Econ. 1998;3(3):319-46.
