# Supplementary material for: A systematic review and quality appraisal of the economic evaluations of schistosomiasis interventions
Source: PLoS Negl Trop Dis. 2022 Oct 12;16(10):e0010822. doi: 10.1371/journal.pntd.0010822 (PMC9591071; doi:10.1371/journal.pntd.0010822)
Supplement: S2 Table — (PDF) [file pntd.0010822.s005.pdf]

S2 Table CHEClist criteria

No.	Criteria	YU et al. (2002) [1]	LESLIE et al. (2011) [2]	CROCE et al (2010) [3]	CARABIN et al. (2000B) [4]	BROOKER et al. (2008) [5]	ZHOU et al. (2005) [6]	YU et al. (2013) [7]	GUO et al. (2005) [8]	GUYATT et al. (2001) [9]
1	Is the study population clearly described?	Y	Y	N	Y	Y	Y	Y	Y	Y
2	Are competing alternatives clearly described?	Y	Y	Y	Y	Y	N	Y	Y	N
3	Is a well-defined research question posed in answerable form?	Y	Y	Y	Y	Y	N	Y	Y	Y
4	Is the economic study design appropriate to the stated objective?	Y	Y	Y	Y	Y	UNCL	Y	Y	Y
5	Is the chosen time horizon appropriate to include relevant costs and consequences	N	Y	Y	Y	Y	Y	UNCL	Y	Y
6	Is the actual perspective chosen appropriate?	Y	N	Y	Y	Y	N	N	N	N
7	Are all important and relevant costs for each alternative identified?	Y	Y	Y	Y	Y	UNCL	Y	N	UNCL
8	Are all the costs measured appropriately in physical units?	Y	Y	Y	Y	UNCL	Y	UNCLEAR	Y	UNCLEAR
9	Are costs valued appropriately?	Y	Y	Y	Y	Y	UNCL	UNCL	UNCL	UNCL
10	Are all important and relevant outcomes for each alternative identified?	Y	Y	Y	UNCL	N	UNCL	UNCL	UNCL	N
11	Are all outcomes measured appropriately?	Y	Y	Y	Y	Y	Y	Y	Y	Y
12	Are outcomes valued appropriately?	Y	Y	Y	Y	Y	Y	Y	Y	Y
13	Is an incremental analysis of costs and outcomes of alternatives performed?	N	N	N	N	N	N	N	N	N
14	Are all future costs and outcomes discounted appropriately?	N	N	N	N	N	N	N	N	N
15	Are all important variables, whose values are uncertain, appropriately subjected to sensitivity analysis?	UNCL	Y	Y	UNCL	Y	N	N	N	N
16	Do the conclusions follow from the data reported?	Y	Y	Y	Y	Y	Y	Y	Y	Y
17	Does the study discuss the generalizability of the results to other settings and patient/client groups?	N	N	N	Y	Y	N	N	N	Y-
18	Does the article indicate that there is no potential conflict of interest of study researcher(s) and funder(s)?	N	Y	N	Y	UNCL	N	UNCL	N	UNCL
19	Are ethical and distributional issues discussed appropriately?	Y	Y	Y	N	Y	N	N	N	UNCL

References:

1. Yu D, Sarol Jr JN, Hutton G, Tan D, Tanner M. Cost-effectiveness analysis of the impacts on infection and morbidity attributable to three chemotherapy schemes against Schistosoma japonicum in hyperendemic areas of the Dongting Lake region, China. China Southeast Asian J. Trop. Med. Public Health. 2002;33(3):441-57.
2. Leslie J, Garba A, Oliva EB, Barkire A, Tinni AA, Djibo A, Mounkaila I, Fenwick A. Schistosomiais and soil-transmitted helminth control in Niger: cost effectiveness of school based and community distributed mass drug administration. PLoS Negl Trop Dis. 2011 Oct 11;5(10):e1326.
3. Croce D, Porazzi E, Foglia E, Restelli U, Sinuon M, Socheat D, et al. Cost-effectiveness of a successful schistosomiasis control programme in Cambodia (1995-2006). Acta Tropica. 2010;113(3):279-84.
4. Carabin H, Guyatt H, Engels D. A comparative analysis of the cost-effectiveness of treatment based on parasitological and symptomatic screening for Schistosoma mansoni in Burundi. Trop. Med. & Int. Health. 2000;5(3):192-202.
5. Brooker S, Kabatereine NB, Fleming F, Devlin N. Cost and cost-effectiveness of nationwide school-based helminth control in Uganda: intra-country variation and effects of scaling-up. Health Policy and Plan. 2008;23(1):24-35.
6. Zhou XN, Wang LY, Chen MG, Wang TP, Guo JG, Wu XH, et al. An economic evaluation of the national schistosomiasis control programme in China from 1992 to 2000. Acta Tropica. 2005;96(2-3):255-65.
7. Yu Q, Zhao GM, Hong XL, Lutz EA, Guo JG. Impact and Cost-Effectiveness of a Comprehensive Schistosomiasis japonica Control Program in the Poyang Lake Region of China. Int. J. Environ. Res. 2013;10(12):6409-21.
8. Guo JG, Cao CL, Hu GH, Lin H, Li D, Zhu R, et al. The role of 'passive chemotherapy' plus health education for schistosomiasis control in China during maintenance and consolidation phase. Acta Tropica. 2005;96(2-3):177-83.
9. Guyatt HL, Brooker S, Kihamia CM, Hall A, Bundy DA. Evaluation of efficacy of school-based anthelmintic treatments against anaemia in children in the United Republic of Tanzania. Bull. World Health Organ. 2001 ;79(8):695-703. PMID: 11545325; PMCID: PMC2566500.
