# Supplementary material for: A systematic review and quality appraisal of the economic evaluations of schistosomiasis interventions
Source: PLoS Negl Trop Dis. 2022 Oct 12;16(10):e0010822. doi: 10.1371/journal.pntd.0010822 (PMC9591071; doi:10.1371/journal.pntd.0010822)
Supplement: S3 Table — (PDF) [file pntd.0010822.s006.pdf]

S3 Table Philips checklist criteria

No.	Criteria	COLLYER et al. (2019) [1]	LO et al. (2016) [2]	LO et al. (2015) [3]	DE NEVE et al. (2018) [4]	CARABIN et al. (2000 A) [5]	NDEFFO-MBAH et al. (2013 A) [6]	NDEFFO-MBAH et al. (2013 B) [7]	LO et al. (2018) [8]	KIRIGIA (1998) [9]
	**STRUCTURE**									
1	Is there a clear statement of the decision problem?	Y	Y	Y	Y	Y	Y	Y	Y	Y
2	Is the objective of the model specified and consistent with the stated decision problem	Y	Y	Y	Y	Y	Y	Y	Y	Y
3	Is the primary decision maker specified?	Y	Y	Y	Y	N	Y	Y	Y	UNCL
4	Is the perspective of the model stated clearly?	Y	Y	Y	Y	N	Y	Y	Y	Y
5	Are the model inputs consistent with the stated perspective	Y	Y	Y	Y	Y	Y	Y	Y	Y
6	Is the structure of the model consistent with a coherent theory of the health condition under evaluation?	Y	Y	Y	Y	Y	Y	Y	Y	Y
7	Are the sources of data used to develop the structure of the model specified?	Y	Y	Y	UNCL	Y	Y	Y	Y	UNCL
8	Are the structural assumptions reasonable given the overall objective, perspective, and scope of the model?	Y	Y	Y	Y	Y	Y	Y	Y	Y
9	Is there a clear definition of the options under evaluation?	Y	Y	Y	Y	Y	Y	Y	Y	Y
10	Have all feasible and practical options been evaluated?	Y	Y	Y	N	Y	Y	N	Y	Y
11	Is there justification for the exclusion of feasible options?	N/A	N/A	N/A	N	N/A	N/A	N	N/A	N/A
12	Is the chosen model type appropriate given the decision problem and specified causal relationships within the model?	Y	Y	Y	N-	Y	Y	Y	Y	UNCL
13	Is the time horizon of the model sufficient to reflect all important differences between the options?	Y	UNCL	UNCL	N	UNCL	UNCL	UNCL	Y	UNCL
14	Do the disease states (state transition model) or the pathways (decision	N/A	N/A	N/A	UNCL	N/A	Y	UNCL	N/A	UNCL

	tree model) reflect the underlying biological process of the disease in question and the impact of interventions?									
15	Is the cycle length defined and justified in terms of the natural history of disease?	N/A	N/A	N/A	N/A	N/A	UNCL	UNCL	N/A	UNCL
	**DATA**									
16	Are the data identification methods transparent and appropriate given the objectives of the model?	Y	Y	Y	UNCL	Y	Y	Y	Y	Y
17	What choices have been made between data sources are these justified appropriately?	UNCL	UNCL	UNCL	UNCL	UNCL	UNCL	UNCL	UNCL	UNCL
18	Where expert opinion has been used are the methods described and justified?	N/A	N/A	N/A	N/A	N/A	N/A	N/A	N/A	Y
19	Is the choice of baseline data described and justified?	UNCL	UNCL	Y	UNCL	Y	Y	Y	Y	N
20	Are transition probabilities calculated appropriately?	N/A	N/A	N/A	UNCL	N/A	UNCL	UNCL	N/A	UNCL
21	Has a half-cycle correction been applied to both costs and outcomes?	N/A	N/A	N/A	UNCL	N/A	UNCL	UNCL	N/A	UNCL
22	If not, has the omission been justified?	N/A	N/A	N/A	UNCL	N/A	UNCL	UNCL	N/A	UNCL
23	Have the methods and assumptions used to extrapolate short-term results to final outcomes been documented and justified?	Y	Y	Y	Y	Y	Y	Y	Y	Y
24	Are the costs incorporated into the model justified?	Y	Y	Y	Y	Y	Y	Y	Y	Y
25	Has the source for all costs been described?	Y	Y	Y	N	Y	Y	Y	Y	N
26	Have discount rates been described and justified given the target decision maker?	Y	Y	Y	Y	UNCL	Y	Y	Y	Y
27	Are the utilities incorporated into the model appropriate?	UNCL	UNCL	Y	UNCL	UNCL	Y	Y	Y	UNCL
28	Is the source of utility weights referenced?	UNCL	UNCL	Y	Y	N	Y	Y	Y	N/A
29	If data have been incorporated as distributions, has the	N/A	N/A	N/A	N/A	N/A	N/A	N/A	N/A	N/A

	choice of distributions for each parameter been described and justified?									
30	If data are incorporated as point estimates, are the ranges used for sensitivity analysis stated clearly and justified?	Y	Y	Y	UNCL	Y	Y	Y	Y	Y
31	Has the heterogeneity been dealt with by running the model separately for different sub-groups?	N/A	N/A	N/A	N	Y	Y	UNCL	N/A	N
32	Have the results been compared with those of previous models and any differences in results explained?	N	Y	Y	Y	N	UNCL	Y	Y	N/A

References

1. Collyer BS, Turner HC, Hollingsworth TD, Keeling MJ. Vaccination or mass drug administration against schistosomiasis: a hypothetical cost-effectiveness modelling comparison. Parasites & Vectors. 2019;12(1).
2. Lo NC, Lai YS, Karagiannis-Voules DA, Bogoch, II, Coulibaly JT, Bendavid E, et al. Assessment of global guidelines for preventive chemotherapy against schistosomiasis and soil-transmitted helminthiasis: a cost-effectiveness modelling study. Lancet Infect. Dis. 2016;16(9):1065-75.
3. Lo NC, Bogoch II, Blackburn BG, Raso G, N'Goran EK, Coulibaly JT, et al. Comparison of community-wide, integrated mass drug administration strategies for schistosomiasis and soil-transmitted helminthiasis: A cost-effectiveness modelling study. Lancet Glob. Health. 2015;3(10):e629-e38.
4. De Neve JW, Andriantavison RL, Croke K, Krisam J, Rajoela VH, Rakotoarivony RA, et al. Health, financial, and education gains of investing in preventive chemotherapy for schistosomiasis, soil-transmitted helminthiases, and lymphatic filariasis in Madagascar: A modeling study. PLoS Negl Trop Dis. 2018;12(12).
5. Carabin H, Chan MS, Guyatt HL. A population dynamic approach to evaluating the impact of school attendance on the unit cost and effectiveness of school-based schistosomiasis chemotherapy programmes. Parasitology. 2000;121:171-83.
6. Ndeffo Mbah ML, Poolman EM, Atkins KE, Orenstein EW, Meyers LA, Townsend JP, et al. Potential Cost-Effectiveness of Schistosomiasis Treatment for Reducing HIV Transmission in Africa - The Case of Zimbabwean Women. PLoS Negl. Trop. Dis. 2013;7(8).

7. Ndeffo Mbah ML, Kjetland EF, Atkins KE, Poolman EM, Orenstein EW, Meyers LA, et al. Cost-effectiveness of a community-based intervention for reducing the transmission of Schistosoma haematobium and HIV in Africa. Proc. Natl. Acad. Sci. U.S.A. 2013;110(19):7952-7.
8. Lo NC, Gurarie D, Yoon N, Coulibaly JT, Bendavid E, Andrews JR, et al. Impact and cost-effectiveness of snail control to achieve disease control targets for schistosomiasis. Proc. Natl. Acad. Sci. U.S.A. 2018;115(4):E584-E91
9. Kirigia JM. Cost-Utility Analysis of Schistosomiasis Intervention Strategies in Kenya. Environ Dev Econ. 1998;3(3):319-46.
