# Supplementary material for: A systematic review and quality appraisal of the economic evaluations of schistosomiasis interventions
Source: PLoS Negl Trop Dis. 2022 Oct 12;16(10):e0010822. doi: 10.1371/journal.pntd.0010822 (PMC9591071; doi:10.1371/journal.pntd.0010822)
Supplement: S4 Table — (PDF) [file pntd.0010822.s007.pdf]

S4 Table iDSI reference case - trial-based economic evaluations

NO.	PRINCIPLES	YU et al. (2002) [1]	LESLIE et al.(2011) [2]	CROCE et al. (2010) [3]	CARABIN et al. (2000b) [4]	BROOKER et al.(2008) [5]	ZHOU et al. (2005) [6]	YU et al. (2013) [7]	GUO et al. (2005) [8]	GUYATT et al. (2001) [9]
1	Is the economic evaluation communicated clearly and transparently to enable decision makers to interpret the methods and results?	INCOMPLETE	YES	INCOMPLETE	INCOMPLETE	INCOMPLETE	INCOMPLETE	INCOMPLETE	INCOMPLETE	YES
2	Do the comparators against which costs and effects are measured accurately reflect the decision problem?	YES	YES	YES	YES	YES	NO	YES	YES	NO
3	Does the economic evaluation consider all the available evidence relevant to the decision problem?	YES	YES	YES	YES	YES	YES	YES	YES	YES
4	Is the measure of health outcome appropriate to the decision problem, should capture positive and negative effects on length of life and quality of life and generalisable across disease states	NO	NO	NO	NO	NO	NO	NO	NO	NO
5	Are all differences between intervention and comparator in expected resource use and costs of delivery to the target population incorporated into the evaluation?	YES	YES	YES.	YES	YES	YES	NO	INCOMPLETE	YES
6	Is the time horizon used in the economic evaluation of a sufficient length to capture all costs and effects relevant to the decision problem, and is an appropriate discount rate used to discount costs and effects to present values	INCOMPLETE	INCOMPLETE	INCOMPLETE	INCOMPLETE	INCOMPLETE	INCOMPLETE	INCOMPLETE	INCOMPLETE	INCOMPLETE
7	Are non-health effects and costs associated with gaining or providing access to health interventions that don't accrue to the health budget identified where relevant to the decision problem. Are all the costs and effects disagregated, either by sector of the economy or to whom they accrue?	INCOMPLETE	NO	YES	INCOMPLETE	YES	NO	NO	NO	NO
8	Are the costs and effects of the intervention on sub-populations within the decision problem explored and the implications appropriately characterised?	NO	YES	NO	NO	NO	NO	NO	NO	NO
9	Is the uncertainty associated with an economic evaluation appropriately characterised?	YES	YES	YES	YES	YES	NO	NO	NO	NO
10	Is the impact of implementing the intervention on the health budget and on other constraints identified clearly and separately?	NO	NO	YES	NO	NO	NO	NO	NO	NO
11	Does the economic evaluation explore the equity implications of implementing the intervention?	NO	NO	YES	NO	YES	NO	NO	NO	INCOMPLETE

References

1. Yu D, Sarol Jr JN, Hutton G, Tan D, Tanner M. Cost-effectiveness analysis of the impacts on infection and morbidity attributable to three chemotherapy schemes against Schistosoma japonicum in hyperendemic areas of the Dongting Lake region, China. China Southeast Asian J. Trop. Med. Public Health. 2002;33(3):441-57.

2. Leslie J, Garba A, Oliva EB, Barkire A, Tinni AA, Djibo A, Mounkaila I, Fenwick A. Schistosomiais and soil-transmitted helminth control in Niger: cost effectiveness of school based and community distributed mass drug administration. PLoS Negl Trop Dis. 2011 Oct 11;5(10):e1326.

3. Croce D, Porazzi E, Foglia E, Restelli U, Sinuon M, Socheat D, et al. Cost-effectiveness of a successful schistosomiasis control programme in Cambodia (1995-2006). Acta Tropica. 2010;113(3):279-84.

4. Carabin H, Guyatt H, Engels D. A comparative analysis of the cost-effectiveness of treatment based on parasitological and symptomatic screening for Schistosoma mansoni in Burundi. Trop. Med. & Int. Health. 2000;5(3):192-202.

5. Brooker S, Kabatereine NB, Fleming F, Devlin N. Cost and cost-effectiveness of nationwide school-based helminth control in Uganda: intra-country variation and effects of scaling-up. Health Policy and Plan. 2008;23(1):24-35.

6. Zhou XN, Wang LY, Chen MG, Wang TP, Guo JG, Wu XH, et al. An economic evaluation of the national schistosomiasis control programme in China from 1992 to 2000. Acta Tropica. 2005;96(2-3):255-65.

7. Yu Q, Zhao GM, Hong XL, Lutz EA, Guo JG. Impact and Cost-Effectiveness of a Comprehensive Schistosomiasis japonica Control Program in the Poyang Lake Region of China. Int. J. Environ. Res. 2013;10(12):6409-21.

8. Guo JG, Cao CL, Hu GH, Lin H, Li D, Zhu R, et al. The role of 'passive chemotherapy' plus health education for schistosomiasis control in China during maintenance and consolidation phase. Acta Tropica. 2005;96(2-3):177-83.

9. Guyatt HL, Brooker S, Kihamia CM, Hall A, Bundy DA. Evaluation of efficacy of school-based anthelmintic treatments against anaemia in children in the United Republic of Tanzania. Bull. World Health Organ. 2001 ;79(8):695-703. PMID: 11545325; PMCID: PMC2566500.
