# Supplementary material for: A systematic review and quality appraisal of the economic evaluations of schistosomiasis interventions
Source: PLoS Negl Trop Dis. 2022 Oct 12;16(10):e0010822. doi: 10.1371/journal.pntd.0010822 (PMC9591071; doi:10.1371/journal.pntd.0010822)
Supplement: S5 Table — (PDF) [file pntd.0010822.s008.pdf]

S5 Table iDSI reference case - decision-analytical models

No.	PRINCIPLES	COLLYER et al. (2019) [1]	LO et al. (2016) [2]	LO et al. (2015) [3]	DE NEVE et al.(2018) [4]	CARABIN et al. (2000a) [5]	NDEFFO-MBAH et al. (2013a) [6]	NDEFFO-MBAH et al. (2013b) [7]	LO et al. (2018) [8]	KIRIGIA (1998) [9]
1	Is the economic evaluation communicated clearly and transparently to enable decision makers to interpret the methods and results?	YES	YES	YES	YES	YES	YES	YES	YES	INCOMPLETE
2	Do the comparators against which costs, and effects are measured accurately reflect the decision problem?	YES	YES	YES	YES	YES	YES	YES	YES	YES
3	Does the economic evaluation consider all the available evidence relevant to the decision problem?	YES	YES	YES	YES	YES	YES	YES	YES	N/A
4	Is the measure of health outcome appropriate to the decision problem, should capture positive and negative effects on length of life and quality of life and generalisable across disease states	NO	YES	YES	YES	NO	NO	YES	YES	YES
5	Are all differences between intervention and comparator in expected resource use and costs of delivery to the target population incorporated into the evaluation?	YES	YES	YES	INCOMPLETE	INCOMPLETE	YES	YES	YES	YES
6	Is the time horizon used in the economic evaluation of a sufficient length to capture all costs and effects relevant to the decision problem, and is an appropriate discount rate used to discount costs and effects to present values	YES	YES	YES	NO	INCOMPLETE	YES	YES	YES	INCOMPLETE

7	Are non-health effects and costs associated with gaining or providing access to health interventions that don't accrue to the health budget identified where relevant to the decision problem. Are all the costs and effects disaggregated, either by sector of the economy or to whom they accrue?	INCOMPL ETE	INCOMPL ETE	INCOM PLETE	YES	NO	INCOMPLE TE	INCOMPLE TE	INCOMPL ETE	YES
8	Are the costs and effects of the intervention on sub-populations within the decision problem explored and the implications appropriately characterised?	YES	YES	YES	NO	YES	NO	NO	YES	NO
9	Is the uncertainty associated with an economic evaluation appropriately characterised?	YES	YES	YES	YES	YES	YES	YES	YES	YES
10	Is the impact of implementing the intervention on the health budget and on other constraints identified clearly and separately?	NO	NO	NO	YES	NO	NO	NO	NO	NO
11	Does the economic evaluation explore the equity implications of implementing the intervention?	NO	NO	NO	NO	YES	NO	NO	NO	NO

References

1. Collyer BS, Turner HC, Hollingsworth TD, Keeling MJ. Vaccination or mass drug administration against schistosomiasis: a hypothetical cost-effectiveness modelling comparison. Parasites & Vectors. 2019;12(1).

2. Lo NC, Lai YS, Karagiannis-Voules DA, Bogoch, II, Coulibaly JT, Bendavid E, et al. Assessment of global guidelines for preventive chemotherapy against schistosomiasis and soil-transmitted helminthiasis: a cost-effectiveness modelling study. Lancet Infect. Dis. 2016;16(9):1065-75.

3. Lo NC, Bogoch II, Blackburn BG, Raso G, N'Goran EK, Coulibaly JT, et al. Comparison of community-wide, integrated mass drug administration strategies for schistosomiasis and

soil-transmitted helminthiasis: A cost-effectiveness modelling study. Lancet Glob. Health. 2015;3(10):e629-e38.

4. De Neve JW, Andriantavison RL, Croke K, Krisam J, Rajoela VH, Rakotoarivony RA, et al. Health, financial, and education gains of investing in preventive chemotherapy for schistosomiasis, soil-transmitted helminthiases, and lymphatic filariasis in Madagascar: A modeling study. PLoS Negl Trop Dis. 2018;12(12).

5. Carabin H, Chan MS, Guyatt HL. A population dynamic approach to evaluating the impact of school attendance on the unit cost and effectiveness of school-based schistosomiasis chemotherapy programmes. Parasitology. 2000;121:171-83.

6. Ndeffo Mbah ML, Poolman EM, Atkins KE, Orenstein EW, Meyers LA, Townsend JP, et al. Potential Cost-Effectiveness of Schistosomiasis Treatment for Reducing HIV Transmission in Africa - The Case of Zimbabwean Women. PLoS Negl. Trop. Dis. 2013;7(8).

7. Ndeffo Mbah ML, Kjetland EF, Atkins KE, Poolman EM, Orenstein EW, Meyers LA, et al. Cost-effectiveness of a community-based intervention for reducing the transmission of Schistosoma haematobium and HIV in Africa. Proc. Natl. Acad. Sci. U.S.A. 2013;110(19):7952-7.

8. Lo NC, Gurarie D, Yoon N, Coulibaly JT, Bendavid E, Andrews JR, et al. Impact and cost-effectiveness of snail control to achieve disease control targets for schistosomiasis. Proc. Natl. Acad. Sci. U.S.A. 2018;115(4):E584-E91

9. Kirigia JM. Cost-Utility Analysis of Schistosomiasis Intervention Strategies in Kenya. Environ Dev Econ. 1998;3(3):319-46.
