# Supplementary material for: A systematic review and quality appraisal of the economic evaluations of schistosomiasis interventions
Source: PLoS Negl Trop Dis. 2022 Oct 12;16(10):e0010822. doi: 10.1371/journal.pntd.0010822 (PMC9591071; doi:10.1371/journal.pntd.0010822)
Supplement: S6 Table — (PDF) [file pntd.0010822.s009.pdf]

S6 Table EMBASE search strategy : 1 January 1998 -17 July 2020

No.	Search Terms	Results
1	(Schistosom* or bilharz* or snail fever).mp. [mp=title, abstract, heading word, drug trade name, original title, device manufacturer, drug manufacturer, device trade name, keyword, floating subheading word, candidate term word]	35788
2	exp Cost-Benefit Analysis/	84384
3	(cost* adj2 (effective* or utilit* or benefit* or consequence* or minim*)).mp. [mp=title, abstract, heading word, drug trade name, original title, device manufacturer, drug manufacturer, device trade name, keyword, floating subheading word, candidate term word]	342396
4	(economic adj2evaluation* or economic* or economic model*).mp. [mp=title, abstract, heading word, drug trade name, original title, device manufacturer, drug manufacturer, device trade name, keyword, floating subheading word, candidate term word]	666078
5	(decision adj (analy* or model* or tree*)).mp. [mp=title, abstract, heading word, drug trade name, original title, device manufacturer, drug manufacturer, device trade name, keyword, floating subheading word, candidate term word]	31062
6	2 or 3 or 4 or 5	943770
7	1 and 6	1341
8	limit 7 to (English language and yr="1998 -Current" and English)	977
