# Supplementary material for: A systematic review and quality appraisal of the economic evaluations of schistosomiasis interventions
Source: PLoS Negl Trop Dis. 2022 Oct 12;16(10):e0010822. doi: 10.1371/journal.pntd.0010822 (PMC9591071; doi:10.1371/journal.pntd.0010822)
Supplement: S7 Table — (PDF) [file pntd.0010822.s010.pdf]

S7 Table Web of Science search strategy : 1 January 1998- 17 July 2020

Number	Search Terms	Results
1	ALL=(schistosom* or bilharz* or snail fever)	19571
2	ALL= (cost benefit* or cost effective* or cost utilit* or cost minim* or cost consequence*)	704428
3	ALL=(economic adj2evaluation* or economic* or economic model*)	1390154
4	ALL= (decision adj (analy* or model* or tree*)).	905146
5	#2 OR #3 OR #4	2739339
6	#1 AND #5	2179
7	(#6) AND LANGUAGE: (English)	2162
