# Supplementary material for: A systematic review and quality appraisal of the economic evaluations of schistosomiasis interventions
Source: PLoS Negl Trop Dis. 2022 Oct 12;16(10):e0010822. doi: 10.1371/journal.pntd.0010822 (PMC9591071; doi:10.1371/journal.pntd.0010822)
Supplement: S8 Table — (PDF) [file pntd.0010822.s011.pdf]

S8 Table MEDLINE and MEDLINE In-process and non-indexed citations search strategy: 1 January 1998- 17 July 2020

Number	Search Terms	Results
1	(Schistosom* or bilharz* or snail fever).mp. [mp=title, abstract, original title, name of substance word, subject heading word, floating sub-heading word, keyword heading word, organism supplementary concept word, protocol supplementary concept word, rare disease supplementary concept word, unique identifier, synonyms]	33366
2	exp Cost-Benefit Analysis/	81071
3	(cost* adj2 (effective* or utilit* or benefit* or consequence* or minim*)).mp. [mp=title, abstract, original title, name of substance word, subject heading word, floating sub-heading word, keyword heading word, organism supplementary concept word, protocol supplementary concept word, rare disease supplementary concept word, unique identifier, synonyms]	168058
4	(economic adj2evaluation* or economic* or economic model*).mp. [mp=title, abstract, original title, name of substance word, subject heading word, floating sub-heading word, keyword heading word, organism supplementary concept word, protocol supplementary concept word, rare disease supplementary concept word, unique identifier, synonyms]	628259
5	(decision adj (analy* or model* or tree*)).mp. [mp=title, abstract, original title, name of substance word, subject heading word, floating sub-heading word, keyword heading word, organism supplementary concept word, protocol supplementary concept word, rare disease supplementary concept word, unique identifier, synonyms]	22349
6	2 or 3 or 4 or 5	723548
7	1 and 6	942
8	limit 7 to (english language and yr="1998 -Current" and english)	576
