# Supplementary material for: A systematic review and quality appraisal of the economic evaluations of schistosomiasis interventions
Source: PLoS Negl Trop Dis. 2022 Oct 12;16(10):e0010822. doi: 10.1371/journal.pntd.0010822 (PMC9591071; doi:10.1371/journal.pntd.0010822)
Supplement: S9 Table — (PDF) [file pntd.0010822.s012.pdf]

S9 Table EconLit search strategy: 1 January 1998- 17 July 2020

Number	Search Terms	Results
1	schistosomiasis AND (economic evaluation or cost-effectiveness or cost-effectiveness evaluation or cost#benefit analysis or economic modelling)	13
