# Supplementary material for: A systematic review and quality appraisal of the economic evaluations of schistosomiasis interventions
Source: PLoS Negl Trop Dis. 2022 Oct 12;16(10):e0010822. doi: 10.1371/journal.pntd.0010822 (PMC9591071; doi:10.1371/journal.pntd.0010822)
Supplement: S10 Table — (PDF) [file pntd.0010822.s013.pdf]

S10 Table - Funding sources declared by authors

No.	Author (Year)	Funding
**1**	Yu et al. (2002) [1]	X
**2**	Leslie et al. (2011) [2]	Donor
**3**	Croce et al. (2010) [3]	X
**4**	Carabin et al. (2000b) [4]	Governmental organisation
**5**	Brooker et al. (2008) [5]	Donor<br>Research/Academic Centre
**6**	Zhou et al. (2005) [6]	Government
**7**	Yu et al. (2013) [7]	Government
**8**	Guo et al. (2005) [8]	X
**9**	Guyatt et al. (2001) [9]	Donor<br>Research/Academic centre<br>NGO
**10**	Collyer et al. (2019) [10]	Donor<br>Research/ Academic centre
**11**	Lo et al. (2016) [11]	Donor<br>Research/Academic centre
**12**	Lo et al. (2015) [12]	Donor<br>Research/Academic centre
**13**	De Neve et al. (2018) [13]	Governmental
**14**	Carabin et al.(2000a) [14]	X
**15**	Ndeffo-Mbah et al. (2013a) [15]	Research/Academic centre
**16**	Ndeffo-Mbah et al. (2013b) [16]	Research/Academic centre
**17**	Lo et al. (2018) [17]	Donor
**18**	Kirigia (1998) [18]	X

<u>References</u>

1. Yu D, Sarol Jr JN, Hutton G, Tan D, Tanner M. Cost-effectiveness analysis of the impacts on infection and morbidity attributable to three chemotherapy schemes against Schistosoma japonicum in hyperendemic areas of the Dongting Lake region, China. China Southeast Asian J. Trop. Med. Public Health. 2002;33(3):441-57.

2. Leslie J, Garba A, Oliva EB, Barkire A, Tinni AA, Djibo A, Mounkaila I, Fenwick A. Schistosomiais and soil-transmitted helminth control in Niger: cost effectiveness of school based and community distributed mass drug administration. PLoS Negl Trop Dis. 2011 Oct 11;5(10):e1326.
3. Croce D, Porazzi E, Foglia E, Restelli U, Sinuon M, Socheat D, et al. Cost-effectiveness of a successful schistosomiasis control programme in Cambodia (1995-2006). Acta Tropica. 2010;113(3):279-84.
4. Carabin H, Guyatt H, Engels D. A comparative analysis of the cost-effectiveness of treatment based on parasitological and symptomatic screening for Schistosoma mansoni in Burundi. Trop. Med. & Int. Health. 2000;5(3):192-202.
5. Brooker S, Kabatereine NB, Fleming F, Devlin N. Cost and cost-effectiveness of nationwide school-based helminth control in Uganda: intra-country variation and effects of scaling-up. Health Policy and Plan. 2008;23(1):24-35.
6. Zhou XN, Wang LY, Chen MG, Wang TP, Guo JG, Wu XH, et al. An economic evaluation of the national schistosomiasis control programme in China from 1992 to 2000. Acta Tropica. 2005;96(2-3):255-65.
7. Yu Q, Zhao GM, Hong XL, Lutz EA, Guo JG. Impact and Cost-Effectiveness of a Comprehensive Schistosomiasis japonica Control Program in the Poyang Lake Region of China. Int. J. Environ. Res. 2013;10(12):6409-21.
8. Guo JG, Cao CL, Hu GH, Lin H, Li D, Zhu R, et al. The role of 'passive chemotherapy' plus health education for schistosomiasis control in China during maintenance and consolidation phase. Acta Tropica. 2005;96(2-3):177-83.
9. Guyatt HL, Brooker S, Kihamia CM, Hall A, Bundy DA. Evaluation of efficacy of school-based anthelmintic treatments against anaemia in children in the United Republic of Tanzania. Bull. World Health Organ. 2001 ;79(8):695-703. PMID: 11545325; PMCID: PMC2566500.
10. Collyer BS, Turner HC, Hollingsworth TD, Keeling MJ. Vaccination or mass drug administration against schistosomiasis: a hypothetical cost-effectiveness modelling comparison. Parasites & Vectors. 2019;12(1).
11. Lo NC, Lai YS, Karagiannis-Voules DA, Bogoch, II, Coulibaly JT, Bendavid E, et al. Assessment of global guidelines for preventive chemotherapy against schistosomiasis and soil-transmitted helminthiasis: a cost-effectiveness modelling study. Lancet Infect. Dis. 2016;16(9):1065-75.
12. Lo NC, Bogoch II, Blackburn BG, Raso G, N'Goran EK, Coulibaly JT, et al. Comparison of community-wide, integrated mass drug administration strategies for schistosomiasis and

soil-transmitted helminthiasis: A cost-effectiveness modelling study. Lancet Glob. Health. 2015;3(10):e629-e38.

13. De Neve JW, Andriantavison RL, Croke K, Krisam J, Rajoela VH, Rakotoarivony RA, et al. Health, financial, and education gains of investing in preventive chemotherapy for schistosomiasis, soil-transmitted helminthiases, and lymphatic filariasis in Madagascar: A modeling study. PLoS Negl Trop Dis. 2018;12(12).
14. Carabin H, Chan MS, Guyatt HL. A population dynamic approach to evaluating the impact of school attendance on the unit cost and effectiveness of school-based schistosomiasis chemotherapy programmes. Parasitology. 2000;121:171-83.
15. Ndeffo Mbah ML, Poolman EM, Atkins KE, Orenstein EW, Meyers LA, Townsend JP, et al. Potential Cost-Effectiveness of Schistosomiasis Treatment for Reducing HIV Transmission in Africa - The Case of Zimbabwean Women. PLoS Negl. Trop. Dis. 2013;7(8).
16. Ndeffo Mbah ML, Kjetland EF, Atkins KE, Poolman EM, Orenstein EW, Meyers LA, et al. Cost-effectiveness of a community-based intervention for reducing the transmission of Schistosoma haematobium and HIV in Africa. Proc. Natl. Acad. Sci. U.S.A. 2013;110(19):7952-7.
17. Lo NC, Gurarie D, Yoon N, Coulibaly JT, Bendavid E, Andrews JR, et al. Impact and cost-effectiveness of snail control to achieve disease control targets for schistosomiasis. Proc. Natl. Acad. Sci. U.S.A. 2018;115(4):E584-E91
18. Kirigia JM. Cost-Utility Analysis of Schistosomiasis Intervention Strategies in Kenya. Environ Dev Econ. 1998;3(3):319-46.
