# Supplementary material for: A systematic review and quality appraisal of the economic evaluations of schistosomiasis interventions
Source: PLoS Negl Trop Dis. 2022 Oct 12;16(10):e0010822. doi: 10.1371/journal.pntd.0010822 (PMC9591071; doi:10.1371/journal.pntd.0010822)
Supplement: S11 Table — (PDF) [file pntd.0010822.s014.pdf]

S11 Table – Hand search exclusions

**INACCESIBLE ARTICLES- ENGLISH**	
1.	Jiang SD, Li XC, Huang FP, et al. Study on cost-effectiveness of clue chemotherapy in hyper-endemic areas of schistosomiasis. Joint Research Management Committee (JRMC) Project Report, Proceeding of JRMC Summary Meeting in Wuhan, China, 1999 (in Chinese) [1].
2.	Lin DD, Jiang QW, Liu YM, et al. Cost-effectiveness analysis of different chemotherapy strategies against schistosomiasis in Poyang Lake region. Zheng QS, Zheng J, editors. Social medicine and schistosomiasis. Tianjin: Tianjin Science and Technology Press, 2000. p. 191-4 (in Chinese) [2].
3	Liu BZ, Wei JB, Wang FH, et al. Cost-effectiveness analysis of different control strategies against schistosomiasis. Zheng QS, Zheng J, editors. Social medicine and schistosomiasis. Tianjin: Tianjin Science and Technology Press, 2000. p. 188-90 (in Chinese) [3].
4	Qiu ZL, Wu X, Chen JX, et al. Cost-effectiveness analysis of optimized strategies for schistosomiasis control. Zheng QS, Zheng J, editors. Social medicine and schistosomiasis. Tianjin: Tianjin Science and Technology Press, 2000. p. 181-3 (in Chinese) [4].
**INACCESSIBLE ARTICLES- CHINESE**	
1	Jiang, SD, Gao, P, Huang, XH., Sangguan, CL. Cost-effectiveness analysis on the project of schistosomiasis control in marshland regions. Chin. J. Schisto. Contr.2002; 14: 385–87 [5].
2	Sun, LP, Zhou, XN, Rong, GR, Wang, YX, Cai, G, Hong, QB, et al. Cost-effectiveness analysis on the project of schistosomiasis control by concreting banks of rivers connecting with the Yangtze River. Chin. J. Schisto. Contr. 2002; 14, 38–43 [6].

References

1. Jiang SD, Li XC, Huang FP, et al. Study on cost-effectiveness of clue chemotherapy in hyper-endemic areas of schistosomiasis. Joint Research Management Committee (JRMC) Project Report, Proceeding of JRMC Summary Meeting in Wuhan, China, 1999 (in Chinese).

2. Lin DD, Jiang QW, Liu YM, et al. Cost-effectiveness analysis of different chemotherapy strategies against schistosomiasis in Poyang Lake region. Zheng QS, Zheng J, editors. Social medicine and schistosomiasis. Tianjin: Tianjin Science and Technology Press, 2000. p. 191-4 (in Chinese).

3. Liu BZ, Wei JB, Wang FH, et al. Cost-effectiveness analysis of different control strategies against schistosomiasis. Zheng QS, Zheng J, editors. Social medicine and schistosomiasis. Tianjin: Tianjin Science and Technology Press, 2000. p. 188-90 (in Chinese).

4. Qiu ZL, Wu X, Chen JX, et al. Cost-effectiveness analysis of optimized strategies for schistosomiasis control. Zheng QS, Zheng J, editors. Social medicine and schistosomiasis. Tianjin: Tianjin Science and Technology Press, 2000. p. 181-3 (in Chinese).

5. Jiang, SD, Gao, P, Huang, XH., Sangguan, CL. Cost-effectiveness analysis on the project of schistosomiasis control in marshland regions. Chin. J. Schisto. Contr.2002; 14: 385–87

6. Sun, LP, Zhou, XN, Rong, GR, Wang, YX, Cai, G, Hong, QB, et al. Cost-effectiveness analysis on the project of schistosomiasis control by concreting banks of rivers connecting with the Yangtze River. Chin. J. Schisto. Contr. 2002; 14, 38–43.
